# Indicator metrics and temporal aggregations introduce ambiguities in water scarcity estimates

**DOI:** 10.1038/s41598-024-65155-5

**Published:** 2024-07-02

**Authors:** Fitsume T. Wolkeba, Mesfin M. Mekonnen, Kate A. Brauman, Mukesh Kumar

**Affiliations:** 1 Department of Civil, Construction, and Environmental Engineering, University of Alabama, Tuscaloosa, AL USA; 2 Global Water Security Center, Alabama Water Institute, University of Alabama, Tuscaloosa, AL USA

**Keywords:** Environmental sciences, Hydrology, Civil engineering

## Abstract

Water scarcity is a global challenge affecting billions of people worldwide. This study systematically assesses differences in the estimation of the global population exposed to water scarcity based on 7 water scarcity indicators and 11 Environmental Flow Requirements (EFR) evaluated at various spatial and temporal resolutions. All indicators show an increase in water scarcity since 1901. However, considering monthly average water scarcity estimates spatially aggregated at the basin scale found 35% less population exposed than estimates based on a distributed grid over the landscape. Estimates temporally disaggregated to consider water scarcity for at least one month a year found 50% (tenfold) larger population exposed compared to average monthly (annual) estimates. The study illustrates that estimates of the impacts of water scarcity are an artifact of how water scarcity is defined and calculated. This suggests caution is needed when relying on a single method and emphasizes the importance of considering the diversity of factors that can influence estimates of impact when assessing water scarcity.

## Introduction

Water scarcity is a growing problem affecting billions of people worldwide, with its severity varying temporally and spatially^1–14^. Achieving water security, having enough water to support consistent and reliable food, energy, health, and livelihoods, is a growing challenge in the face of population growth and increased water use over the last century^15^. Recent meetings and initiatives, such as the United Nations 2023 Water Conference and Sustainable Development Goal 6, reflect the concern of leaders and policymakers. Understanding water scarcity, including how it varies over space and time, is crucial for developing effective policies and management strategies.

Previous studies have assessed water scarcity using various indicators, models, scales, and input data, resulting in estimates ranging from 0.5 billion^12^ to 4.3 billion^16^ people affected. The range of estimates reflects differences in the definition and calculation of water scarcity, choice of indicators, spatial and temporal resolution, method of calculating environmental flow requirement (EFR), and model and input data used^10,13,17–24^. A summary of these papers, providing different water scarcity evaluation indicators and configurations, along with the respective number of people exposed to water scarcity, is presented in Table S1.

The choice of indicator used to measure water scarcity is a critical factor in determining the estimated number of people exposed to water scarcity. These indicators evaluate the relationship between water use and availability and define water scarcity based on a predefined threshold. The most common indicators include (1) water availability per capita using the Falkenmark index^1,5,9,21,25–27^, (2) withdrawal to availability (WTA) ratio^6,12,17,20,21^, (3) cumulative withdrawal to demand (CWD) ratio^20,28^, (4) consumption to low-flow Q90 ratio^20^, (5) consumption to availability ratio^13,18,19,29^, and (6) consumption to availability ratio after accounting for EFR^6,12,13,15,16,29–31^. Water availability per capita (Falkenmark index; 1) is a less data intensive, easy to use, and intuitive population-driven indicator, but its thresholds do not account for regional variations in demand, and it does not consider external factors such as water import, infrastructure, and efficiency. WTA (2) is usually applied at the annual scale and so indiciates water stress based on annual withdrawal and water availability data and thus lacks information on the sub-annual distribution of water shortage. Consumption to availability (5) and withdrawal to water availability (2) ratios indicate demand-based water scarcity and can be applied in smaller spatial and temporal scales but require substantial data and modeling to acquire input data. Consumption or withdrawal to water availability ratios that account for EFR (6) account for the flow required for sustaining the ecosystem. CWD (3) accounts for the sub-annual distribution of water availability and use, but it is not designed to differentiate between water scarcity triggered by a single major event and those caused by frequent minor occurrences^20,21,28^. Finally, the consumption to low-flow Q90 ratio (4) accounts for the seasonal availability of water, but it is best applied in low-flow conditions^20^. A detailed theoretical comparison of most of these water scarcity indicators can be found in Hussain et al.^22^ and Hanasaki et al.^20^.

The temporal and spatial resolution at which water scarcity is calculated is also important. Increased temporal resolution provides insights into the seasonality and frequency of water scarcity and can help in assessing trends. As water scarcity can vary significantly throughout the year due to seasonal fluctuations in precipitation, temperature, reservoir operation, and water demand, higher temporal resolution allows for a more accurate tracking of scarcity. Furthermore, a higher temporal resolution can aid in response planning at emerging scarcity hotspots, and optimize resource allocation^32^. Studies have assessed the temporal resolution at monthly^16,18,19,29,33^ and annual scales^5,6,12,13,16,17,19,34^. Increased spatial resolution provides more granular insight into the geographical extent of water scarcity. A higher spatial resolution is useful to perform localized assessment, and develop targeted interventions such as infrastructure development, water conservation programs, and policy measures that address unique needs of each region. The most common spatial resolutions at which water scarcity is assessed are at the country scale^6,12^, by basin^6,18,19,33^, and evenly distributed across the landscape in a grid^6,11,16,17,29^.

Environmental Flow Requirements (EFR) describe the amount of water needed to sustain freshwater ecosystems^31,34–36^. Water scarcity assessments consider this water unavailable for human use. By reducing the water available for human use, EFR increases the ratio of withdrawals or consumption to available water. The number of people experiencing water scarcity ranged from 2.0 billion to 4.0 billion depending on the EFR method used^29^.

Regardless of how water scarcity is defined, different models and input data, each with distinct assumptions and preparation procedures, affect estimates of water scarcity^10,37^. Model intercomparison projects have shown substantial variation in water availability and water use estimates among models^10,15,38,39^. Similarly, different data sources affect estimates of water availability and water use^40–50^.

Because differences in estimates of water scarcity can stem from all of these sources, the extent to which differences among indicators affect estimates of water scarcity is ambiguous. In the past, researchers have attempted to understand the individual influences of indicators, resolution, and environmental flows on water scarcity by reviewing previous works^9,22,23,51–55^ or by selecting a specific number of indicators at certain temporal and spatial resolutions^20,21,28,29^. Here, we disentangle the effects of different indicators, resolutions, and EFRs by using the same model and data to compare variation in estimates of water scarcity.

## Materials and methods

**Figure 1:**
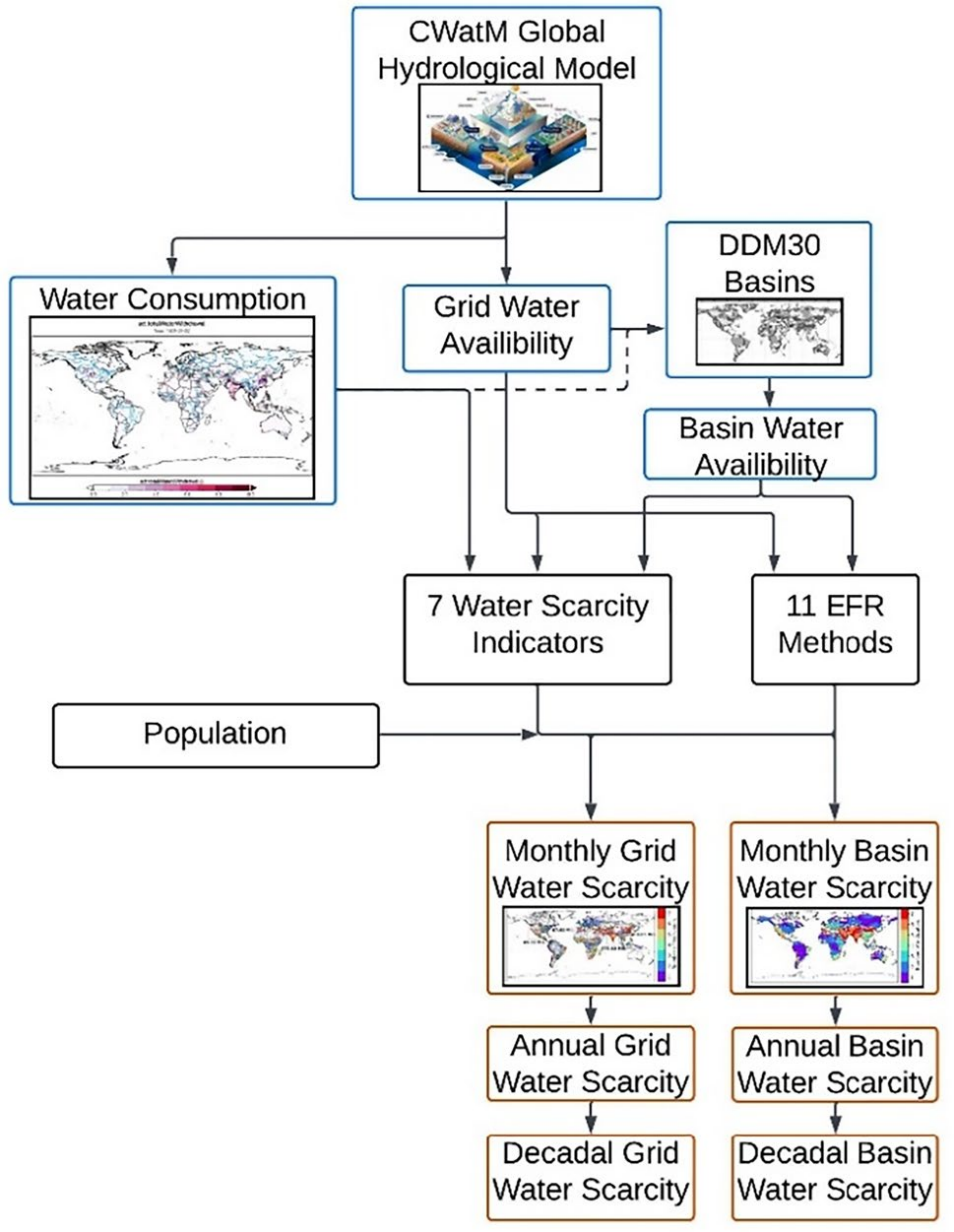
Schematic showing workflow.

The ensuing analysis was conducted following the approach outlined in Fig. 1. We used a single model, the Community Water Model (CWatM), and consistent data to estimate water availability and consumption distributed across the landscape in a grid-based model. We calculated water scarcity using 7 water scarcity indicators and 11 EFR methods. We then used a basin boundary map to aggregate up to total water consumption, water availability, and water scarcity at the basin level. Finally, we incorporated population data to estimate the number of people exposed to above-moderate water scarcity in both distributed and basin spatial levels given different temporal resolutions.

### Data and hydrologic model

Our analysis employed a state-of-the-art global hydrologic model, CWatM. CWatM is an open-source gridded hydrological and channel routing model^56^. We ran the model on a daily timestep at 0.5-degree resolution from 1871 to 2019, with the first 30 years used for model spin-up using data provided by the International Institute for Applied Systems Analysis (IIASA)^56^ for CWatM. Other datasets included GRDC station data^57^, global drainage direction map (DDM30) basin boundary^58^, climate data (W5E5) (Cucchi,^59^; Lange,^60^), and population data^61^. Additional details on data sources used in our analysis are in Tables S4 and S5. Details of the data used in the CWatM model are found at IIASA^62^. Due to limitations in data availability, we held landcover data constant after 2010 and before 1960 and we used crop coefficient data from the year 2000 for all years, consistent with CWatM^56^.

CWatM is capable of ingesting both net and gross water demand inputs from sectors such as domestic, livestock, and industry. Additionally, the model calculates the net demand for irrigation water. The distinction between gross and net demand is crucial. Gross demand is the total water demand, including the volume of water that will be returned. Net demand is the subset of gross demand that is used or evaporated and will not be returned.

Within each 0.5-degree grid at which CWatM runs, it calculates abstraction, withdrawal, and consumption. Total abstraction is the water extracted from surface water, including channels and reservoirs, and groundwater, including renewable and fossil aquifers, to satisfy gross demand. A portion of this water is returned to the source; consumption is water that is not returned, representing net demand. Both withdrawal and consumption are quantified for irrigation, domestic, livestock, and industry. The Total withdrawal refers to the amount of available water required to meet the gross demand from these sectors. Consumption refers to the amount of water that is extracted by these sectors and subsequently evaporated into the atmosphere, thus not returning to the system. In essence, consumption represents the net demand (withdrawal minus return flow) that can be met with the available water.

The CWatM model offers two operational modes. One mode limits water abstraction to the available renewable groundwater, while the other mode does not impose any limitations on the available groundwater, thereby using nonrenewable fossil groundwater. When there is sufficient renewable water to meet all gross demand, total abstraction, total withdrawal, and total gross demand are all equal. In this scenario, total consumption will also be equal to total net demand. However, when the available water is insufficient to meet the gross demand, it results in unique relationships among these variables. In such situations, nonrenewable fossil water is utilized. Consequently, total abstraction is equal to the total available renewable water. Total withdrawal matches total abstraction, which is less than the total gross demand. Finally, total consumption is calculated as total abstraction minus return flow.

When calculating discharge distributed across the grid, The CWatM accounts for evaporation from the channel, water extraction from the channel, return flow within the grid, and net inflow from the upstream grid. Further details about the model can be found in Burek et al.^56^.

We ran the model twice (Fig. 1), once considering sectoral demand incorporating reservoirs, and once only considering natural lakes and without accounting for water use. The first model incorporated 3663 reservoirs from the Hydrolakes database^39,56,63,64^. Incorporating reservoirs attenuates seasonal water variability. The model also computes evaporation from reservoirs. Since the second model was run without water use or artificial reservoirs, it provides a simulation of natural conditions; EFR was estimated using the results from this model run (Fig. 1).

We assessed model performance using discharge data from more than 10,000 GRDC stations^57^ spanning from 1931 to 2019 using Kling-Gupta Efficiency (KGE)^65^. KGE balances correlation, bias, and the coefficient of variation^66–68^. Monthly KGE values were greater than 0 and 0.5 at 1986 and 552 stations, respectively (Figure S3). It is important to note that this evaluation is not just limited to GRDC gages that follow standardized streamflow measurement protocols thus ensuring consistency of comparisons. Previous studies have validated CWatM at GRDC gages, resulting in KGE greater than 0 at 243 stations out of 1366 considered^56^. CWatM has been compared with other Global Hydrological Models (GHMs), and the results show comparable performance^39^. It is important to acknowledge that performance of all GHMs are affected by factors such as poor forcing data quality, simpler representation of dominant hydrological processes, and poor parameterization. Despite these challenges, GHMs are crucial for global scale assessments^38,69,70^.

### Water scarcity indicators

We assessed global water scarcity using the Falkenmark index, withdrawal to availability (WTA) ratio^6,12,17,20,21^, cumulative withdrawal to demand (CWD) ratio, and consumption to availability ratio (with and without accounting for EFR). Population within regions where indicators were calculated as moderate and above were used for comparison (Table S2). To evaluate trends in water scarcity from 1901 onwards, water scarcity using 80% of natural flow as EFR and another with no EFR consideration was used. We used an EFR of 80% of monthly natural flow for the trend assessment because it has been widely used in previous studies^16,33^. To facilitate the comparison among the various estimates of the number of people experiencing water scarcity from 2010 to 2019, the median of hydrological EFR methods was used in cases where water scarcity was estimated with EFR consideration to account for variation between EFR methods. Details of the EFR methods are summarized in Table S3.

### Spatial and temporal resolution

Water scarcity was estimated at different temporal resolutions (monthly and annual) and spatial scales (grid and basin) using CWatM run on a daily timestep and then aggregated to monthly and annual values. We assessed water scarcity using three temporal units: water scarcity at least one month a year (derived by estimating if the location was exposed to water scarcity for more than one month in a given year), monthly average water scarcity (derived by averaging the monthly water scarcities), and annual water scarcity (derived using annual water use and water availability). The annual water scarcity estimates were used in addition to the monthly estimates, as they are still being used and may provide useful information^71,72^.

The monthly average water scarcity was computed by averaging the monthly values of the water scarcity indicator over a year. The total number of people in water scarcity was obtained by adding up the populations of all water-scarce grids. For estimating water scarcity at least one month a year, the number of months under water scarcity was first estimated. Grids that experienced water scarcity for at least one month a year were selected, and the annual population for each grid was counted. Any population within a grid identified as experiencing water scarcity during at least one month a year was counted as facing water scarcity. The study compared the impacts on the number of people and land area affected by water scarcity at decadal intervals.

The study used a grid resolution of 0.5 degrees and 10,832 basin boundaries defined based on a global drainage direction map, DDM30 basins^58^. There are several options for basin aggregations. In this study, DDM30 Basins were chosen because they align with CWatM’s use of DDM30 flow direction maps for flow direction definition, and their boundaries have been previously validated in literature for global modeling applications^58^. In addition, all DDM30 basins outlet either to the ocean or inland sink, reducing inter-basin interactions that may affect water availability calculations.

To estimate water scarcity at different spatial scales, the grid was considered to be a hydrological unit and the water scarcity indicator was calculated using grid-scale water availability and consumption*.* Total water availability at the grid level was calculated by adding the runoff generated within the grid (before it is used within the grid cell) to the upstream net inflow (upstream runoff minus upstream water use) and then subtracting the EFR. Basin-level water scarcity was calculated by aggregating consumption over the entire basin and water availability was calculated by subtracting the EFR from the natural flow at the outlet of the basin. The basins used in this study do not have inlets.

To compare spatial variation among the indicators, we used grid-based and monthly estimations of the number of people and land area under water scarcity from 2010 to 2019. We selected grid-based and monthly estimates because they are the finer spatial and temporal scales commonly used^13,16,20,21,28^.

## Results

### Trends in water scarcity using monthly and grid estimates

**Figure 2:**
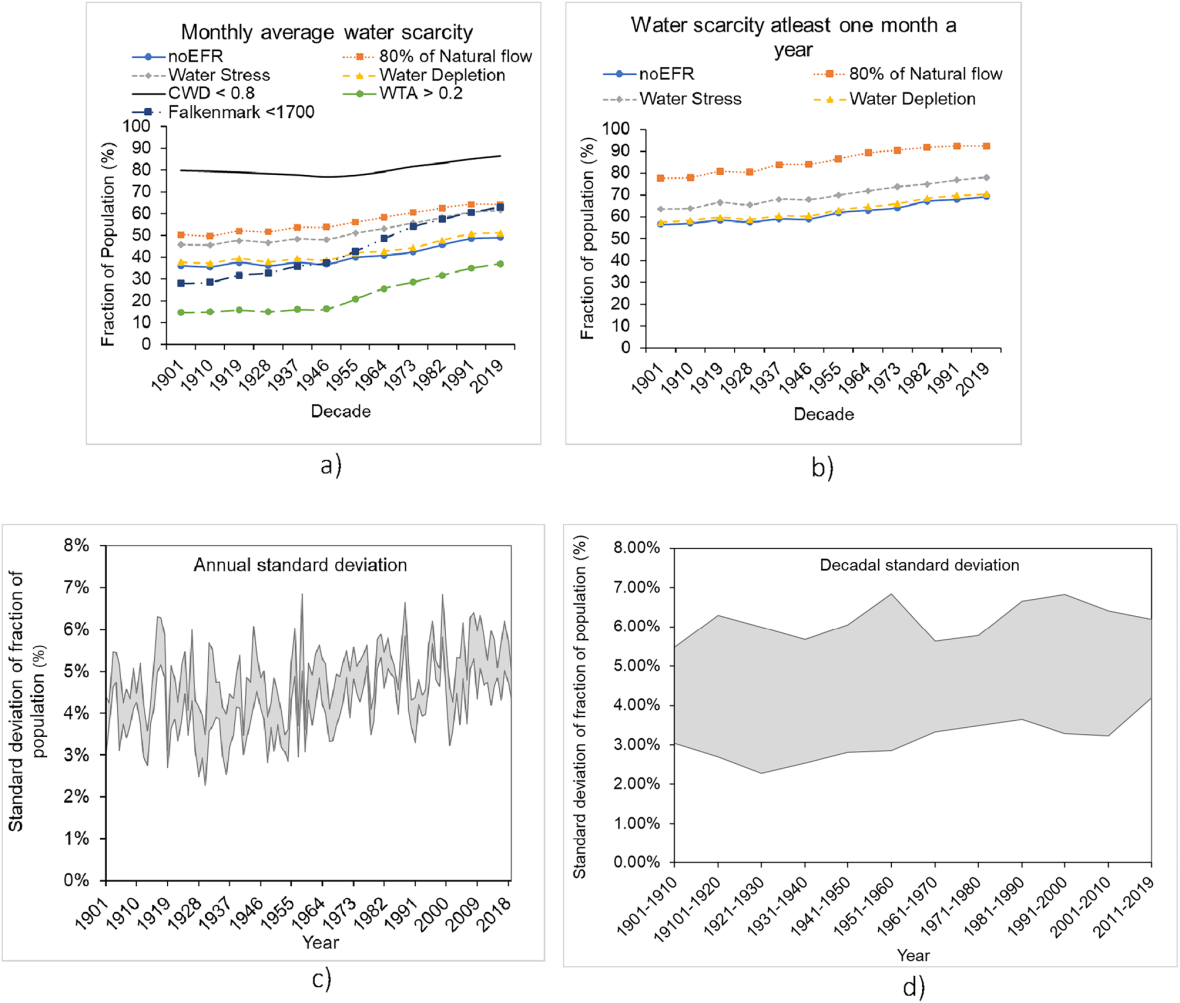
Water scarcity trends 1901–2019. Water scarcity considering no EFR and 80% of natural flow as EFR, water stress, and water depletion. (**a**) Monthly average water scarcity (**b**) Water scarcity at least one month a year (CWD, WTA, and Falkenmark values are the same for both plots and therefore are shown only in a), standard deviation ranges between water stress, water depletion, and water scarcity considering no EFR, and 80% of natural flow as EFR (**c**) annual standard deviation ranges, and (**d**) decadal standard deviation ranges.

We found that, for all indicators, the number of people facing water scarcity consistently increased in each decade since 1901 (see Fig. 2). The intra-annual and climatic variability in water scarcity is shown as the annual and the decadal standard deviation ranges (2% to 7%) in Figs. 2c and d. Water scarcity expands geographically over the same period (see Fig. 4). Thus, both the number of people in water-scarce places has increased and the number of water scarce places has increased. These findings agree with previous estimates^15^ made using a smaller number of indicators. The rate of increase in the number of people experiencing water scarcity has increased since 1950 (Fig. 2, S6, and S7). This trend coincides with the upward trajectory of both population and water use. This effect is particularly noticeable using the Falkenmark indicator because this method directly reflects population.

The number of people estimated to be facing water scarcity for at least one month a year, both as an absolute value and as a fraction of total population, exceeds estimates derived from monthly average water scarcity estimates by 50% on average (Figure S8 and Figure S9). This suggests that the experience of water scarcity may more substantial than indicated by the annual or monthly average values.

### Impact of including EFR in water scarcity estimates

**Figure 3:**
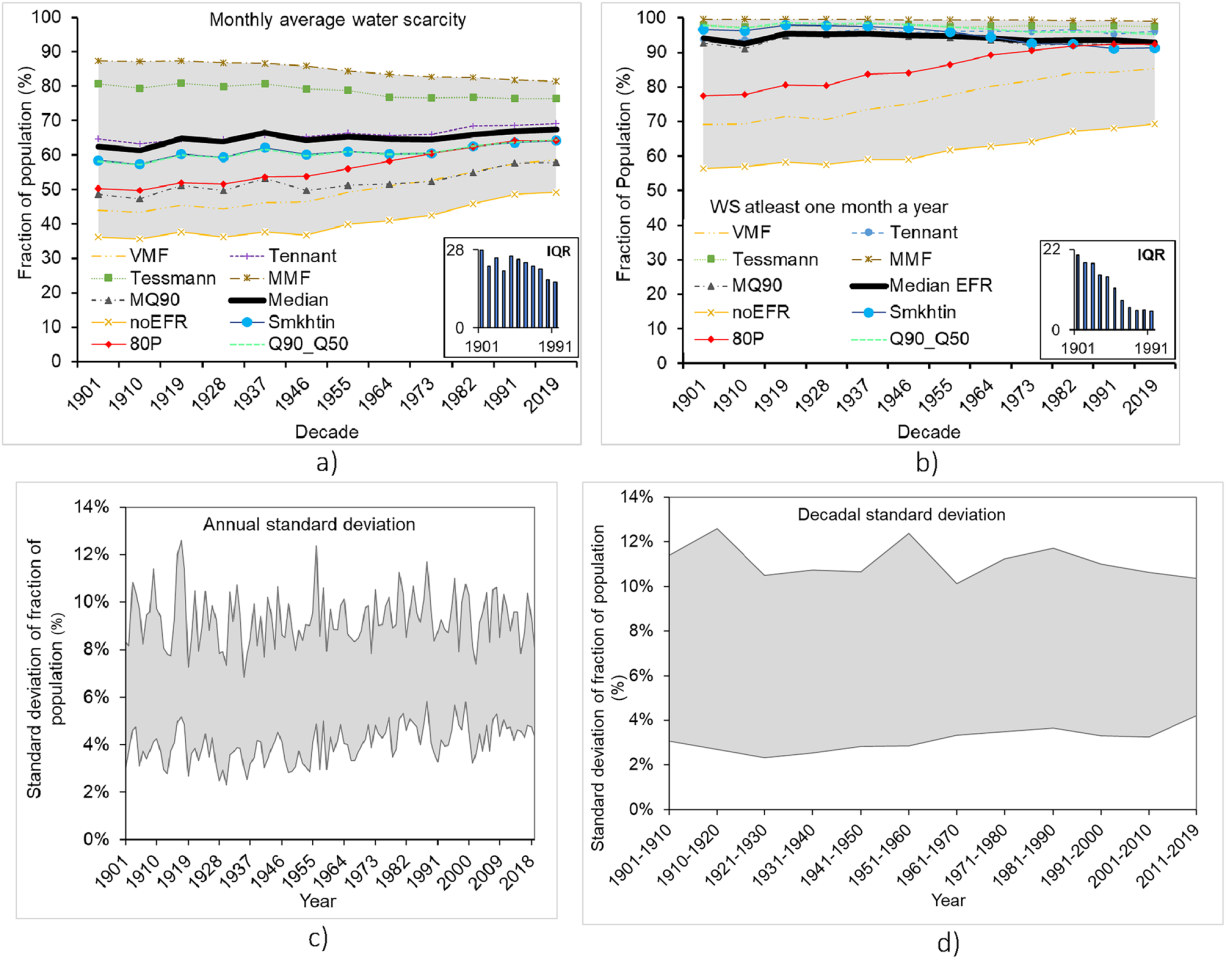
Comparison between the estimated number of people exposed to water scarcity using different EFR methods. Variable monthly flow (VMF), Tessmann, MQ90, no EFR, 80% of natural flow, Tennant, mean monthly flow (MMF), Smakhtin, Q90_Q50, and median EFR. (**a**) Monthly average water scarcity, (**b**) Water scarcity at least one month a year, (**c**) annual standard deviation ranges, and (**d**) decadal standard deviation ranges.

To evaluate the effect of different methods of estimating EFR on estimates of grid-level water scarcity, we calculated the monthly water consumption-to-availability ratio accounting for EFR. We selected this indicator because it facilitates the use of different EFRs. Distinctive variations in water scarcity emerged depending on how the EFR is computed (Figs. 3, S1, and S2). The annual standard deviation and the decadal standard deviation ranges (2% to 13%) are shown in Figs. 3c and d. This underscores the critical role that EFR plays in shaping and influencing the assessments of water scarcity, revealing the nuanced impact of environmental considerations on water availability for human use. When using the long-term mean monthly flow (MMF) as EFR, the number of people exposed to water scarcity on a monthly scale was the highest (Table S6). However, this EFR method is less commonly used, primarily because it allocates nearly all the available natural flow to EFR during low-flow periods. Instead, most EFR methods define EFR as a certain percentage of MMF or the mean annual flow^29,31^. Another method that yielded a high number of people affected by water scarcity was the Tessmann method, which allocates larger percentages of MMF compared to the other methods, leading to higher water scarcity estimates^29,31^.

The number of people affected by water scarcity after accounting for EFR has steadily increased over each successive decade (Figure S3). Temporal scale matters however: The monthly average approach revealed a consistent increase in fraction of population experiencing water scarcity, but when water scarcity at least one month a year is used, the Tessmann, Tennant, Smakhtin, Q90_Q50, MMF, and MQ90 EFR methods, decrease slightly with time (Fig. 3). This decrease in the fraction of people affected reflects a lower rate of increase in population exposed compared to total population growth. This is because the methods consistently estimated a larger fraction of population facing water scarcity in every decade as compared to the other methods. Methods that allocate a larger fraction of water for EFR, such as MMF and Tessmann, exhibited the smallest percentage difference between monthly average and at-least-one month-a-year water scarcity estimates. These methods tend to estimate more people facing water scarcity because they allocate a larger fraction of water for EFR, resulting in less sensitivity to changes in the indicator. In contrast, methods like no EFR and MQ90 that allocate little to no water for EFR resulted in the highest percentage difference between monthly average water scarcity and water scarcity at least one month a year (Figure S9). This suggests that the presence or absence of EFR considerations substantially influences the estimates, with approaches assigning a larger fraction of available water for EFR exhibiting more stability in their results, while those assigning little or no water for EFR show greater variability.

There is less variation among estimates of population facing water scarcity, indicated by a narrower interquartile range (IQR), when calculated for at least one month annually. The spread between estimates as a percentage of the median when using monthly average water scarcity was larger (23%) than when using water scarcity at least one month a year (12%) (Fig. 3a and b). The use of monthly EFR methodologies to assess water scarcity for at least one month a year resulted in a closely clustered estimation (Fig. 3). This clustering is due to a larger fraction of people estimated under water scarcity, making it less sensitive to changes. VMF and no EFR are outliers from this clustering. VMF allocates a smaller fraction of MMF (60%) as EFR during low-flow months, while no EFR does not allocate water for EFR.

### Impact of spatial and temporal resolution on water scarcity estimates

#### Spatial variation

**Figure 4:**
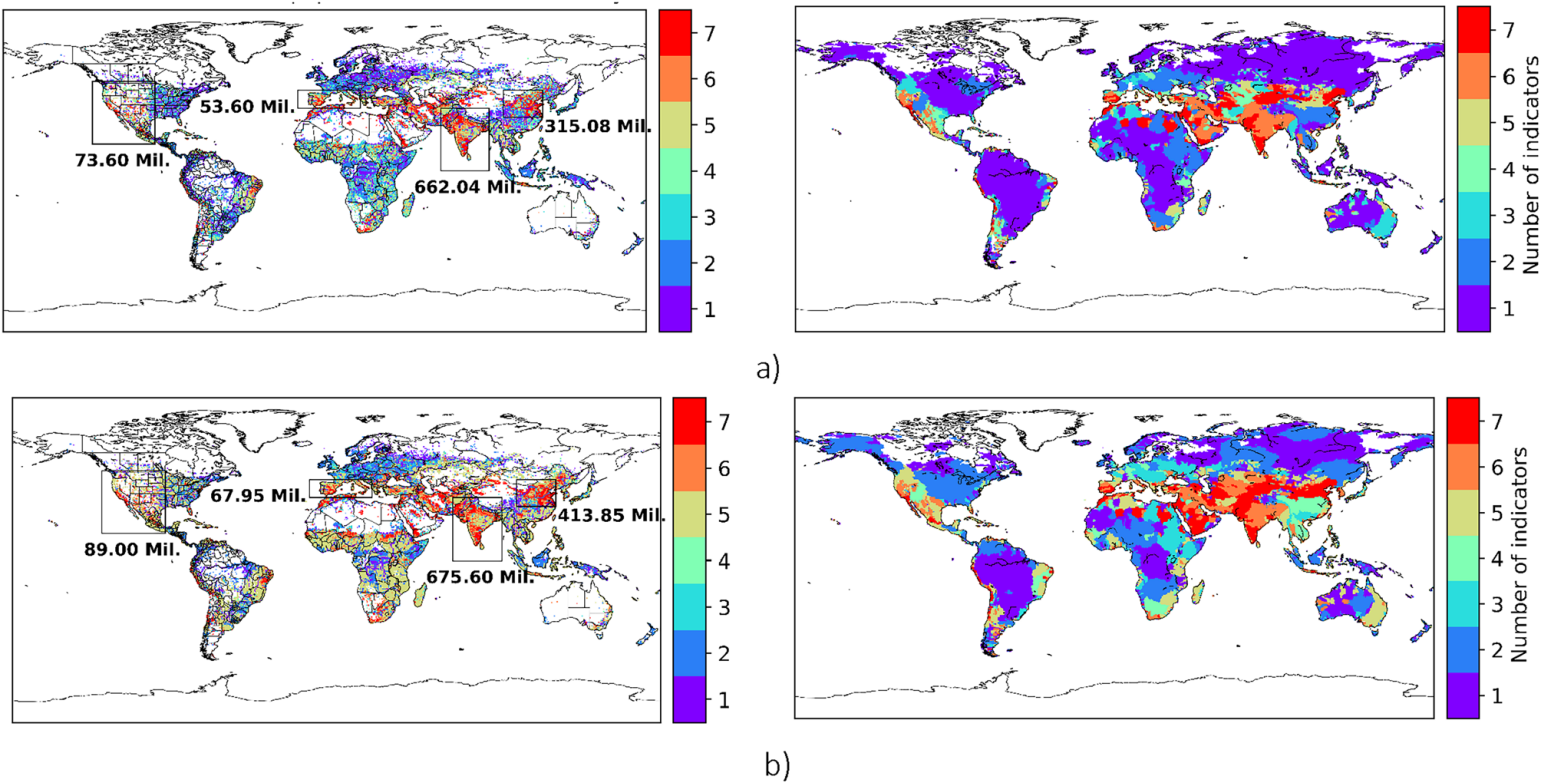
Grid-level (left) and Basin-level (right) distribution of the number of indicators showing moderate or worse water scarcity when scarce location is identified using: (**a**) monthly average water scarcity metric, and (**b**) water scarcity on an average of at least one month a year. Boxes and corresponding number of people indicate population under water scarcity in all seven indicators within the box. Grids with at least one person per square km are shown.

The spatial distribution of water scarcity differs depending on the water scarcity indicator (Fig. 4, right plots). CWD and median EFR based water scarcity estimation result in the highest land area affected and WTA resulted in the lowest land area affected for both monthly average water scarcity and water scarcity at least one month a year.

**Figure 5:**
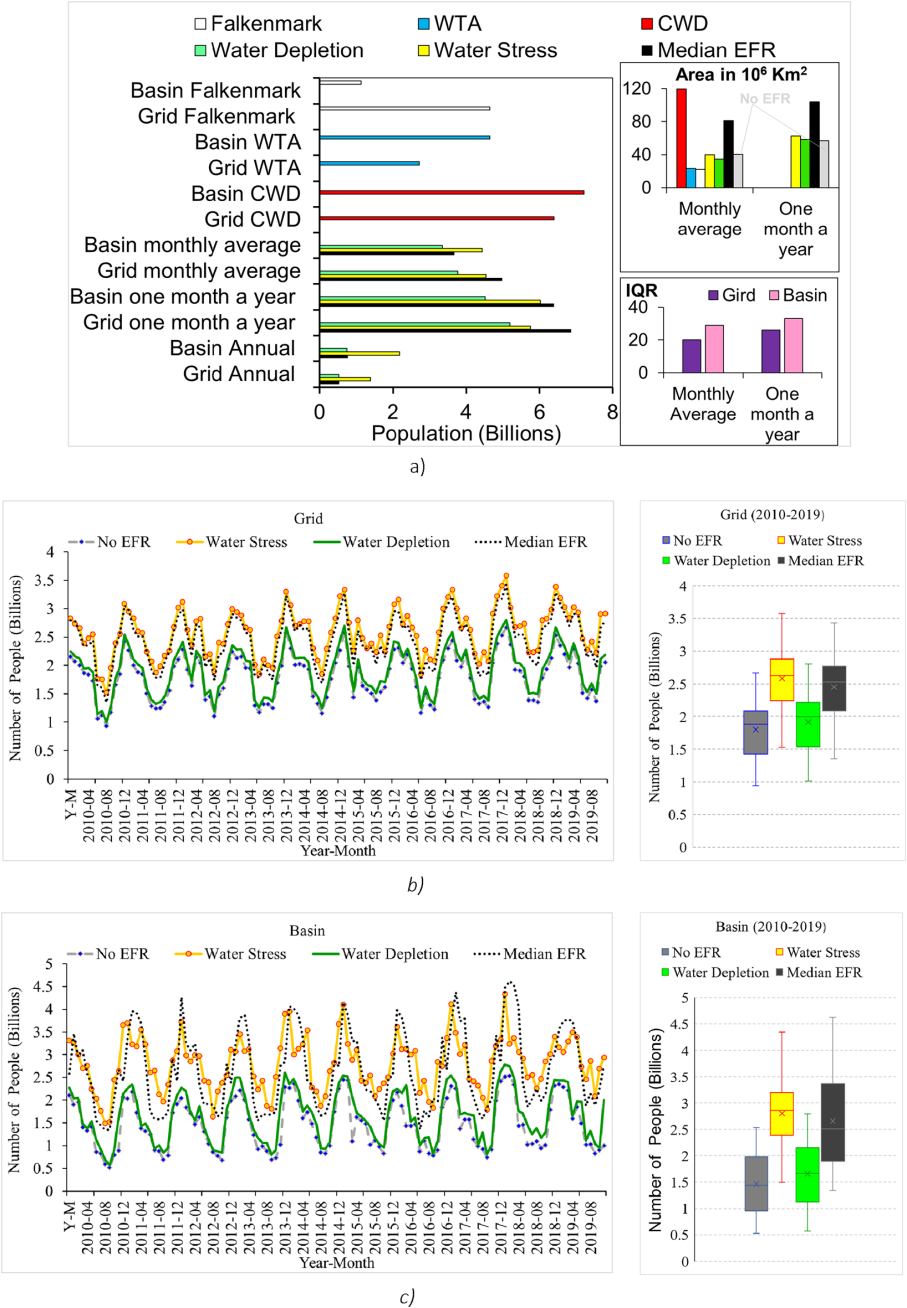
Comparison between different water scarcity indicators over varied temporal and spatial resolutions during 2010–2019. (**a**) The number of people (in billions) exposed to water scarcity (left), and area under water scarcity in million square kilometers (top-right). IQR between monthly average and at least one month a year water scarcity estimates at basin and grid levels (bottom-right) (**b**) Grid level monthly time series (left) and range (right) of number of people exposed to monthly water scarcity 2010–2019, (**c**) Basin level time series (left) and range (right) of number of people exposed to monthly water scarcity 2010–2019.

Despite the difference among the indicators, similar regions are found to be affected, specifically India, southern Europe, the western United States, and Central Asia (Figures 4 and 5). Many of these locations have been previously identified as water-scarce due to the overconsumption of renewable water resources Figs. 4, 5^5,6,13,16,18^

#### Temporal resolution

The number of people exposed varies depending on the temporal scale at which water scarcity is assed, monthly or annual. Across water scarcity indicators, on average (Table S6), annual estimates find more than ten times fewer people facing water scarcity at both the gird and basin levels compared to monthly estimates. This finding is consistent with previous studies^5,9,13,16,18,19^. Additionally, the results showed more variation in annual estimates compared to monthly estimates (Table S6). Monthly estimates consistently identified all major water scarce locations, making them less sensitive to indicator changes and effectively reducing the spread between estimates. Annual estimates identified fewer locations and were more sensitive to changes in indicators because one indicator may identify locations missed by others.

Monthly estimates take into account the seasonal variation in monthly water availability and demand, identifying water shortage during part of the year^16^. The monthly variations are plotted using time series and box plots (Fig. 5). This study used two widely used monthly water scarcity approaches—monthly average water scarcity and water scarcity at least one month a year. However, the minimum, maximum, percentile, or standard deviation of water scarcity estimates can provide useful information about the sub-annual distribution of water scarcity in locations where water scarcity is highly seasonal. Plots of mean, min, max, and standard deviation of water scarcity in basins show the variation in monthly estimates (Figure S11).

The results show that, at a grid level, from 2010 to 2019, the number of people estimated to be facing water scarcity at least one month a year is, on average, 40% larger than the estimate for monthly average water scarcity (Figs. 4, 5, and S8). Using monthly average values, all seven indicators find, between 2010 and 2019, about 1.2 billion people experienced water scarcity. This figure increased to 1.9 billion when estimating water scarcity for at least one month a year (Fig. 4). When using the six water use based indicators (excluding the Falkenmark index), the number of people under water scarcity increased to 1.5 billion when using monthly average estimates and further increased to 2.5 billion when using water scarcity estimated at least one month a year. This underscores, once again, the significance of temporal considerations in assessing water scarcity and highlights the substantial impact on the estimated population facing water stress.

Temporal resolution also matters at the basin scale, at which estimates differed by nearly 70%, depending on whether monthly average or water scarcity at least one month a year was used. Notably, basins such as the Colorado, Murray Darlin, and Yellow River had more indicators signal water scarcity at least one month a year than for monthly average water scarcity (Fig. 4). This implies that the choice of temporal scale significantly influences the identification of water scarcity in specific basins. Water scarcity at least one month a year and monthly average water scarcity estimates agreed in basins located in India, southern Europe, the Western United States, and Central Asia. Comparison to annual temporal resolution is more stark: monthly average basin estimates were 6 times higher than annual estimates and water scarcity at least one month a year was 10 times higher. This substantial difference emphasizes the impact of temporal considerations on basin-level water scarcity assessments, providing insights into the dynamic nature of water availability and demand over different time scales.

Using monthly average water scarcity and water scarcity at least one month a year, basin estimations were, on average, 35% and 13% less than grid estimates, respectively. Using annual estimates of water scarcity, basin estimations were, on average, 20% higher than the grid level (Fig. 5).

Water stress index estimation resulted in a lower percentage of the difference between different spatial resolutions (grid and basin) and temporal resolutions (monthly and annual) (Figures S8 and 6). At the grid level, the Falkenmark index estimates a similar number of people experiencing water scarcity with a monthly average water stress index over the decade (2010–2019). Compared to gridded estimates, the number of people facing water scarcity at the basin scale using the Falkenmark indicator decreased by about threefold (Fig. 5). The cumulative withdrawal to demand ratio (CWD) yielded a larger number of people experiencing water scarcity compared to the other indicators, both at the grid and basin levels, except when assessing grid water scarcity for at least one month a year. In this specific case, the use of the median EFR led to the largest estimate of people experiencing water scarcity.

## Conclusions and discussion

This is the first study to quantitatively assess estimates of population and land area facing water scarcity when calculated using different indicators, different EFR considerations, and different spatial and temporal resolutions using the same data and model. In doing so, we show the extent of variation among the various combinations of indicators, EFR, and spatial and temporal resolutions. We also show the locations and the number of people that indicators agree are facing water scarcity.

Annual estimates tend to obscure the monthly variation in water scarcity, consistently yielding estimates more than ten times lower than monthly values. This discrepancy is particularly pronounced in regions characterized by large intra-annual variability in water scarcity, where monthly estimates provide a more refined picture of water scarcity dynamics. The practice of taking the monthly average of water scarcity introduces a level of abstraction, masking the monthly-scale variations by providing an overall average. This approach does not account for the specific number of months people are subject to water scarcity. As a result, estimates using water scarcity at least one month a year were consistently larger than monthly average estimates by about 50%. The choice between the two monthly methods should align with specific objectives. In naturally water-stressed areas such as arid and hyper-arid regions, where water shortage for one or more months can have consequential effects, using water scarcity at least one month a year might provide more insight. However, monthly average water scarcity is useful for tracking locations where water scarcity occurs more frequently. This underscores the importance of selecting an appropriate temporal scale based on the specific goals of the study.

Grid-level estimates account for water scarcity at a finer resolution and are useful for local assessments. However, grid cell analysis presupposes that processes within the grid cell are well known, which may not be as simple as understanding what’s going on within a basin. So, while estimates at the grid-level may appear to be more precise, they may be misleading. Basin-level studies provide meaningful insights applicable on a tangible scale^9,13,18,19,34^. However, they hide water scarcity at finer resolutions. Any water availability or water use variations at the basin level may lead to a misleading conclusion for the population living within the basin. This discrepancy arises because basin-level estimates assume uniform spatial distribution of water availability and water use. In so doing, basin estimates average out spatial highs and lows in water scarcity, providing an average value that may not accurately represent the extremes. This is particularly relevant considering that the upstream population might not face the same water scarcity as downstream residents, who generally experience the cumulative water use of everyone upstream^73^. Grid-level estimates were generally greater than basin-level estimates by up to 35%. This variation is reversed when using annual water scarcity estimates, WTA, and CWD, where basin estimates were larger by about 20%. Previous studies have also reported a similar higher number of people at the basin level compared to the grid level when in annual estimations^6^. On monthly estimates, the results show a larger spread in basin-level estimations than in grid-level estimations. The larger IQR on the basin level by 20% and 60% when using the monthly average and water scarcity at least one month a year, respectively, is because any changes in the basin level estimates affect the population living in the basin, unlike grid level estimates, where the change only affects the grid population, which is generally smaller than the basin population. The choice of which level to use depends on the intended objective and data availability.

The results also show that the choice of the EFR method affects the estimated number of people under water scarcity (IQR between 12% and 23% of the median). This sensitivity is more pronounced in monthly approaches, particularly when employing methods that allocate less water to EFR. This underscores the importance of carefully weighing the implications of the EFR methods used and tailoring their application to the specific context and goals of the water scarcity assessment. The finding is consistent with previous works that showed water scarcity estimates vary if different EFR is used^29^.

Among the compared indicators, water stress Index exhibited relatively lower sensitivity to spatial and temporal resolution changes. This method estimates a smaller percentage of differences between grid and basin, as well as between monthly average water scarcity and water scarcity at least one month a year. One contributing factor to this is the application of a lower threshold (> 0.2) to the resulting water scarcity estimate. This contrast is evident when considering the use of no EFR with a larger threshold (> 1), which resulted in the highest sensitivity in most cases. The CWD indicator stood out as the only indicator that consistently indicated water scarcity in most geographical areas (Figs. 4 and 5). It also estimated the largest land area under water scarcity, followed by water scarcity considering median EFR (Fig. 5).

Generally, CWD tends to estimate higher water scarcity^20^. This indicator assumes that if the withdrawal from the river is less than 80% of the demand, the location is deemed moderately scarce^20,28^. This implies that if over 20% of the demand is directly satisfied from sources other than river flow (either from reservoirs or groundwater pumping), the area is considered experiencing water scarcity. This interpretation holds true in many cases, given that most irrigation, domestic, and industrial uses rely on either water stored in reservoirs or groundwater pumping. Annual water scarcity, accounting for median EFR, estimated the lowest number of people affected. This can be attributed to the method using annual consumption and water availability, reflecting a distinct approach that considers a broader temporal scale.

While there are several water scarcity indicators available, we chose a subset for this study and did not include noteworthy ones such as the IWMI economic water scarcity^74^, poverty index^75^, and quantity-quality environmental flow requirement (QQE)^76^. These indicators were excluded because they differ inherently from the ones considered here^22^. For instance, IWMI economic water scarcity incorporates economic factors alongside water availability, reflecting issues related to inadequate infrastructure for accessing water resources. The water poverty index considers factors such as time and distance for resource access, institutional capacity, and water availability. QQE includes water quality in addition to quantity.

The exclusion of these indicators was intentional as the focus here primarily revolves around water availability and use. However, future studies could benefit considerably from incorporating these omitted indicators alongside with the ones examined in this study. Such an inclusive approach would provide a more comprehensive understanding of how water scarcity estimations vary depending on factors such as infrastructure, water accessibility, and water quality. It would offer additional insights beyond water availability, contributing to a more holistic assessment of water scarcity. These indicators would help address issues of people’s lived experiences of water scarcity, which this study does not address. Future efforts directed toward understanding this aspect, such as the Household Water Insecurity Experiences scale^77^, would greatly improve water scarcity assessments by providing insights into the real-world implications and challenges faced by communities.

While overall comparisons of water scarcity remain largely consistent regardless of data, the estimated number of people and land affected by water scarcity has varied across studies depending on the data used^10,15,38,39,42–50,56^. Factors identified as important include quality of climate data^44^,Wolkeba & Mekonnen, 2023), crop data, irrigation water withdrawal^78^, reservoir operation ^63,79,80^, and groundwater contributions^81^. To address this issue, future research could focus on evaluating the sensitivity of water scarcity estimates given a range of input data sources. Such assessments would be instrumental in reducing uncertainties related to data and enhancing the reliability of the findings. As datasets evolve or as new and improved approaches for assimilating data emerge, it becomes crucial to update the results.

The evaluation performed here relies on a single global hydrologic model. To enhance confidence in the conclusions drawn, it would be beneficial to apply alternative models under similar configurations of scarcity indices, EFRs, and simulation resolutions.

An important takeaway from this study is that we have highlighted the risk associated with relying on a single method to assess water scarcity. It is crucial to understand the implications of the selected indicator if only one will be used. This study’s findings support presenting a spectrum of outputs that includes a range in the number of people exposed to water scarcity, as well as the various EFR methods, spatial and temporal resolutions, and indicators used. This approach not only clarifies differences between estimates but also allows for the appropriate selection of approaches tailored to specific objectives.

In essence, the study emphasizes the importance of adopting a comprehensive perspective when assessing water scarcity, considering the diversity of factors that can influence the results. This inclusive approach contributes to a more robust and informed decision-making process in water resource management.

### Supplementary Information

Supplementary Information.

## Data Availability

The datasets used and analyzed during the current study are available from the corresponding author upon reasonable request.
