# Supplementary material for: Indicator metrics and temporal aggregations introduce ambiguities in water scarcity estimates
Source: Sci Rep. 2024 Jul 2;14:15182. doi: 10.1038/s41598-024-65155-5 (PMC11219772; doi:10.1038/s41598-024-65155-5)
Supplement: Supplementary file 1 — Supplementary Information. [file 41598_2024_65155_MOESM1_ESM.docx]

*Nature Communications*

Supporting Information for

**Differences in Global Water Scarcity Estimates are an Artifact of How it is Defined and Calculated**

**Fitsume T. Wolkeba^1^, Mesfin M. Mekonnen1, Kate A. Brauman^2^, Mukesh Kumar^1^**

Department of Civil, Construction, and Environmental Engineering, University of Alabama, Tuscaloosa, AL, USA

Global Water Security Center, Alabama Water Institute, University of Alabama, Tuscaloosa, AL, USA

**Contents of this file**

Tables S1-S6

Figure S1-S9

Table S1: Summary of previous studies and reports

| **Study** | **EFR**^[[1]](#footnote-2)^ | **Reference Year of analysis** | **Resolution** | **Population exposed in Billions** | **Metric** |
| --- | --- | --- | --- | --- | --- |
| **UN (2021)** | **-** |  | Country | 2.3 (Water stressed)  0.733 (Critical) |  |
| **UNICEF (2021)** |  |  | Country | 1.42 |  |
| **Liu et al. (2021)** | 60% | 2001-2010 | Grid | ≈2.2(34%) | Rws^[[2]](#footnote-3)^>0.4 |
|  | Q50 |  |  | ≈4.01(61%) | WSI^[[3]](#footnote-4)^>1 |
|  | Tessman |  |  | ≈3.29(50%) | WSI>1 |
|  | Q90 |  |  | ≈2.3(35%) | WSI>1 |
|  | Tennant |  |  | ≈2 (30%) | WSI>1 |
|  | Smakhtin |  |  | ≈2.3(35%) | WSI>1 |
|  | VMF |  |  | ≈2 (31%) | WSI>1 |
|  | Q90/Q50 |  |  | ≈2.4(36%) | WSI>1 |
| **Fao (2020)** |  |  | Irrigation area | 3.2 (high to very high)  1.2 (Severe) |  |
| **Degefu et al. (2018)** | 80% |  | Country-Basin Mesh | 1.6 | WSI>2 |
| **Mekonnen and Hoekstra (2016)** | 80% | 1996-2005 | Grid | 4.3 (71%) | WSI>1 |
| **Alcamo et al (2007)** | 60% | 1995 | Grid | 2.3 | Rws>0.4 |
| **Oki and Kanae (2006)** | 60% | 2000 | Grid | 2.4 | Rws>0.4 |
| **Wada et al. (2011)** | 40% | 2000 | Grid | 1.7-1.8 | Rws>0.4 |
| **Smakhtin et al. (2004)** | 60% | 1961-1990 | Basin | 1.4 | Rws>0.5 |
| **Alcamo et al. (2003)** | 60% | 1995 | Grid | 2.1 | Rws>0.4 |
| **Oki et al. (2001)** | 60% | 1995 | Grid | 1.7 | Rws>0.4 |
|  | 60% | 1995 | Country | 2.2 | Rws>0.4 |
|  | 60% | 1995 | Basin | 2.7 | Rws>0.4 |
| **Vorosmarty et al. (2000)** | 60% | 1995 | Grid | 1.8 | Rws>0.4 |
|  | 60% | 1995 | Country | 0.5 | Rws>0.4 |
| **Kummu et al. (2010)** | 60% | 2005 | Food Production Unit | 2.3 | <1000m3/sec |
| **Islam et al. (2007)** | 60% | 2000 | Grid | 1.8-3.1 |  |
| **Alcamo et al. (2007)** | 60% | 1995 | Grid | 1.6 |  |
| **Arnell (2004)** | 60% | 1995 | sub-basin | 1.4 |  |

Table S2: Water Scarcity Indicators Used

| *Water scarcity Indicator* | Method | Description |
| --- | --- | --- |
| *(Falkenmark, 1997, 2013; Falkenmark et al., 1989)*  *Falkenmark* | *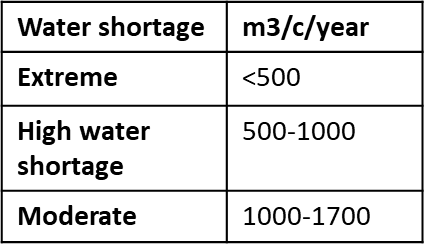* | Also called Water shortage  Annual Index |
| Mekonnen and Hoekstra (2016)  Water Scarcity  $\mathbf{=}\frac{\text{Consumption }}{\begin{aligned} \mathbf{Total water availibility} \\ \mathbf{after accounting for EFR} \end{aligned}}$  $\text{ }$ | 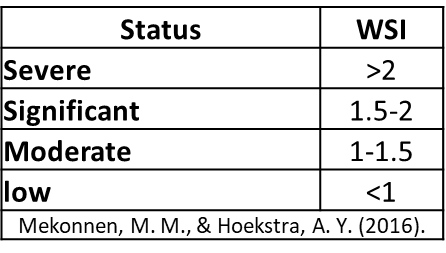 | WS_g_ = water scarcity at a grid  WS_b_= water scarcity of basin  Total Water availability = water availability + net water inflow from upstream cell  = Q _grid_ – EFR + channel abstraction- return flow  $\text{Blue water footprint=}\text{Consumption}\text{ }$  Allows incorporation of different EFR |
| Brauman et al. (2016)  Water Depletion  WD = $\frac{\text{Consumption }}{\mathbf{Total water availibility}}$ | 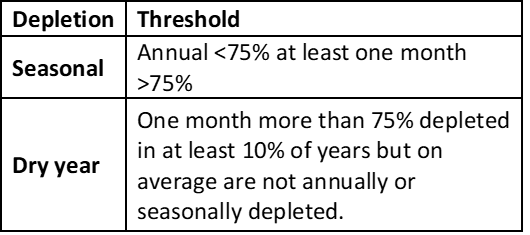 | 75% threshold considered in the current study.  Water depletion is water shortage that affects water supply to demand and environment. It is included in this study because it provides a threshold between water stress index and Water scarcity without considering EFR. |
| Wada et al. (2011)  Water stress index  WSI = $\frac{\text{Consumption }}{\mathbf{Total water availibility}}$ | \| **Water Stress** \| **Threshold** \| \| --- \| --- \| \| **Debilitating** \| >0.8 \| \| **Severe** \| > 0.4 \| \| **Moderate** \| 0.2-0.4 \| \| **Low Water** \| 0.1-0.2 \| \| **No Water Stress** \| <0.1 \| | Assumes some percentage of available water is not accessible for use. |
| Hanasaki et al. (2008)  Cumulative withdrawal to demand ratio (CWD) =$\frac{\sum_{\mathbf{Y}_{\mathbf{start}}}^{\mathbf{Y}_{\mathbf{end}}} \sum_{\mathbf{DOY=1}}^{\mathbf{365}} \mathbf{w}_{\mathbf{Y,DOY}}}{\sum_{\mathbf{Y}_{\mathbf{start}}}^{\mathbf{Y}_{\mathbf{end}}} \sum_{\mathbf{DOY}}^{\mathbf{365}} \mathbf{d}_{\mathbf{Y,DOY}}}$ | \| **CWD** \| **Threshold** \| \| --- \| --- \| \| **Low/no stress** \| CWD > 0.8 \| \| **Medium Stress** \| 0.8<CWD<0.5 \| \| **High stress** \| CWD<0.5 \| | WY= actual water withdrawn from river^[[4]](#footnote-5)^  DY=Gross demand from irrigation, industry, livestock and domestic |
| Hanasaki et al. (2018) Withdrawal to availability (WTA) =$\frac{\mathbf{A}_{\mathbf{ALL,y,i}}}{\mathbf{Q}_{\mathbf{NAT,y,i}}}$ | \| **WTA** \| **Threshold** \| \| --- \| --- \| \| **Low** \| **>0.1** \| \| **Moderate** \| **>0.2** \| \| **High** \| **>0.4** \| \| **Very High** \| **>1** \| | $A_{ALL,y,i}$= total water withdrawal in the ALL simulation (m3/s)  $Q_{NAT,y,i}$ = annual river discharge in the natural simulation (m3/s) including baseflow, respectively |

Table S3: Used Environmental Flow Requirement Methods (More detail can be found (Pastor et al., 2014)

| Method | Scale | Ecological status | EFR consideration |
| --- | --- | --- | --- |
| Tennant (1976) | Monthly /Annual | fair | LFR^[[5]](#footnote-6)^ and HFR^[[6]](#footnote-7)^ |
| Smakhtin et al. (2004) | Monthly/ Annual | fair | LFR and HFR |
| VMF  Pastor et al. (2014) | Monthly/ Annual | fair ecological status | LFR IFR^[[7]](#footnote-8)^ and HFR |
| Richter et al. (2012) | Monthly / Annual | good | 80% of the natural runoff is allocated as an environmental flow requirement. Richter et al. (2012) suggested that around 80% or higher magnitude of daily flow could be required to help preserve the integrity of ecology in most rivers. |
| Q90_Q50  Pastor et al. (2014) | Monthly/ Annual | fair | Q90 for LFR and Q50 for HFR |
| Tessmann (1980) | Monthly | did not define | LFR, IFR and HFR |
| MMF | Monthly |  | Mean Montly flow |
| MQ90 | Monthly |  | Monthly flows equaled or exceeded 90 % of the time |
| MAF | Annual |  |  |
| Q90 | Annual |  | Annual flows equaled or exceeded 90 % of the time |
| Q50 | Annual |  | Annual flows equaled or exceeded 50 % of the time |

Table S4: Sample of the data used in CWatM model.

| Data | Dataset name |
| --- | --- |
| Demand | - Domestic, Industrial and livestock |
| Forest | Forest land cover is used from from (Hansen et al., 2013) |
| Sealed Urban area or impervious surface area | Based on 1km version of (Elvidge et al., 2007) |
| Crop Coefficient | MIRCA2000—Global data set of monthly irrigated and rainfed crop areas around the year 2000. http://www.uni-frankfurt.de/45218023/MIRCA (Portmann et al., 2010) |
| Soil | From the HWSD the standard soil properties like texture, porosity, soil minerals (% of sand, clay), organic matter and bulk density are used. For example, Bulk density second soil layer 5-30 cm depth. pedotransfer is used (Zhang & Schaap, 2017) |
| Lake and reservoirs | The HydroLakes database http://www.hydrosheds.org/page/hydrolakes (Lehner et al., 2011; Messager et al., 2016) |
| Drainage Direction Map | 0.5° drainage direction map (DDM30) of Doll and Lehner (2002) |
| Source:, additional data on all the datasets used in CWatM model can be found at [https://cwatm.iiasa.ac.at/data.html#](https://cwatm.iiasa.ac.at/data.html) | |

Table S5: Description of additional data used.

| Data | Dataset name | Source |
| --- | --- | --- |
| Climate data | - GSWP3-W5E5 observational climate input data for ISIMIP3a  - Spin up data from 1971-1900 | ISIMIP3a (2021) |
| Population | - ISIMIP3a population data | ISIMIP3a (2022) |
| Global Basins | - 10,832 DDM30 basins. Outlet of basins is either sink or the ocean | Doll and Lehner (2002) |
| GRDC Discharge | - Monthly flow Data (1931-2019) from 10,703 GRDC discharge stations. Original provided monthly flow is used and where there is none GRDC estimated monthly flow is used. | GRDC (2007) |

Table S6: Decadal average of number of people (Billions) exposed to water scarcity (Water scarcity is computed in places where there is population > 1)

|  |  |
| --- | --- |

| Both the grid and basin level estimations in 2010–2019 show that most monthly methods indicate there is above-average water scarcity in the Godvari, Ganges, and Krishna basins in India; Shatt Al Arab in Iraq; the Luan He and Liao He in the Yellow Sea; the Hai He basins in the East China Sea; the Guadalquivir and Guadiana basins in Europe; the basins on the West Coast of the US, including Sacramento, Concepcion, Yaqui, and the Fuerte Nuevo; the Gascoyne and Fortescue River basins in Australia; and the Gouritz, Olifants, and Barakat basins in Africa. Some of these basins are heavily populated (Figures 1 and 2).  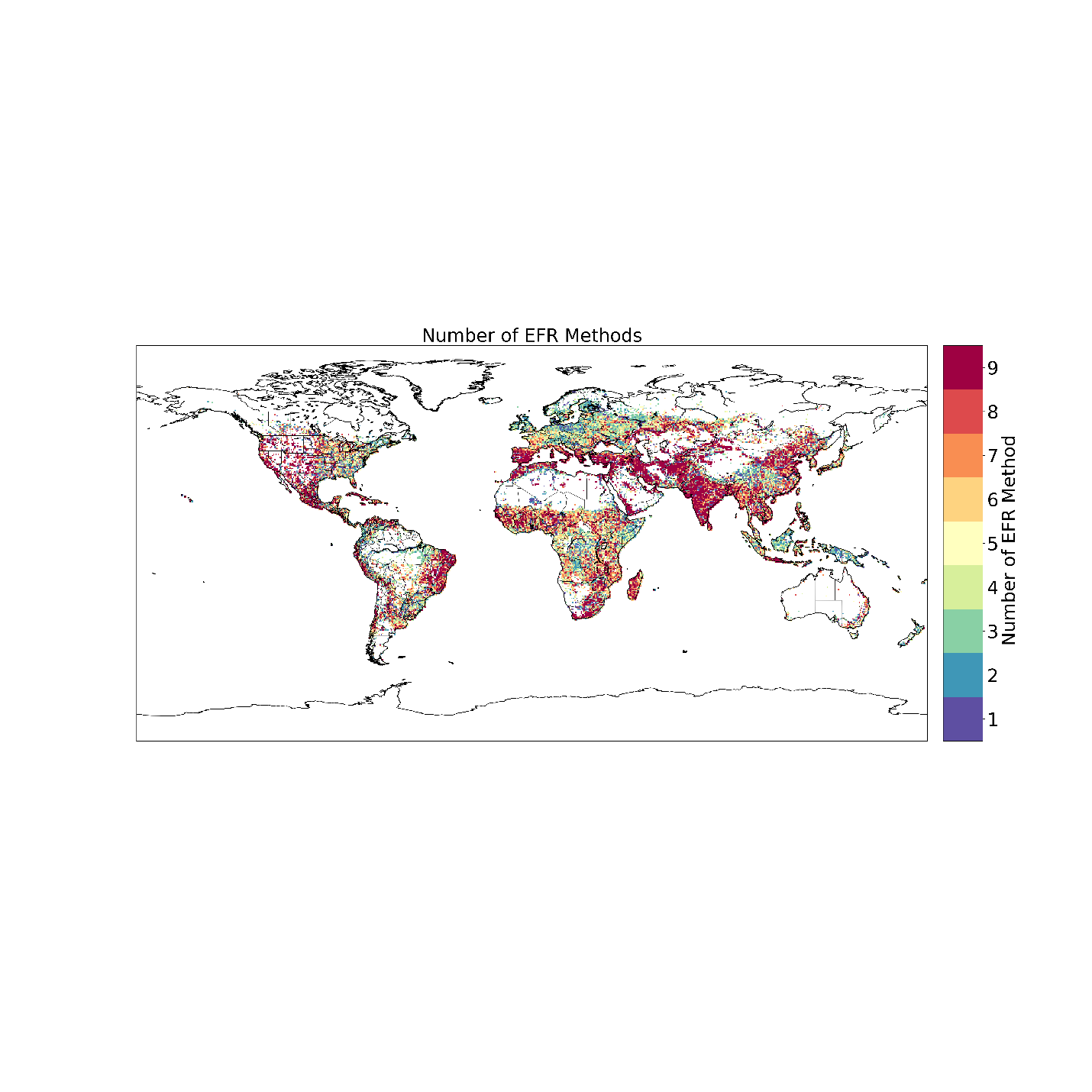   1. average of monthly water scarcity |
| --- |
| 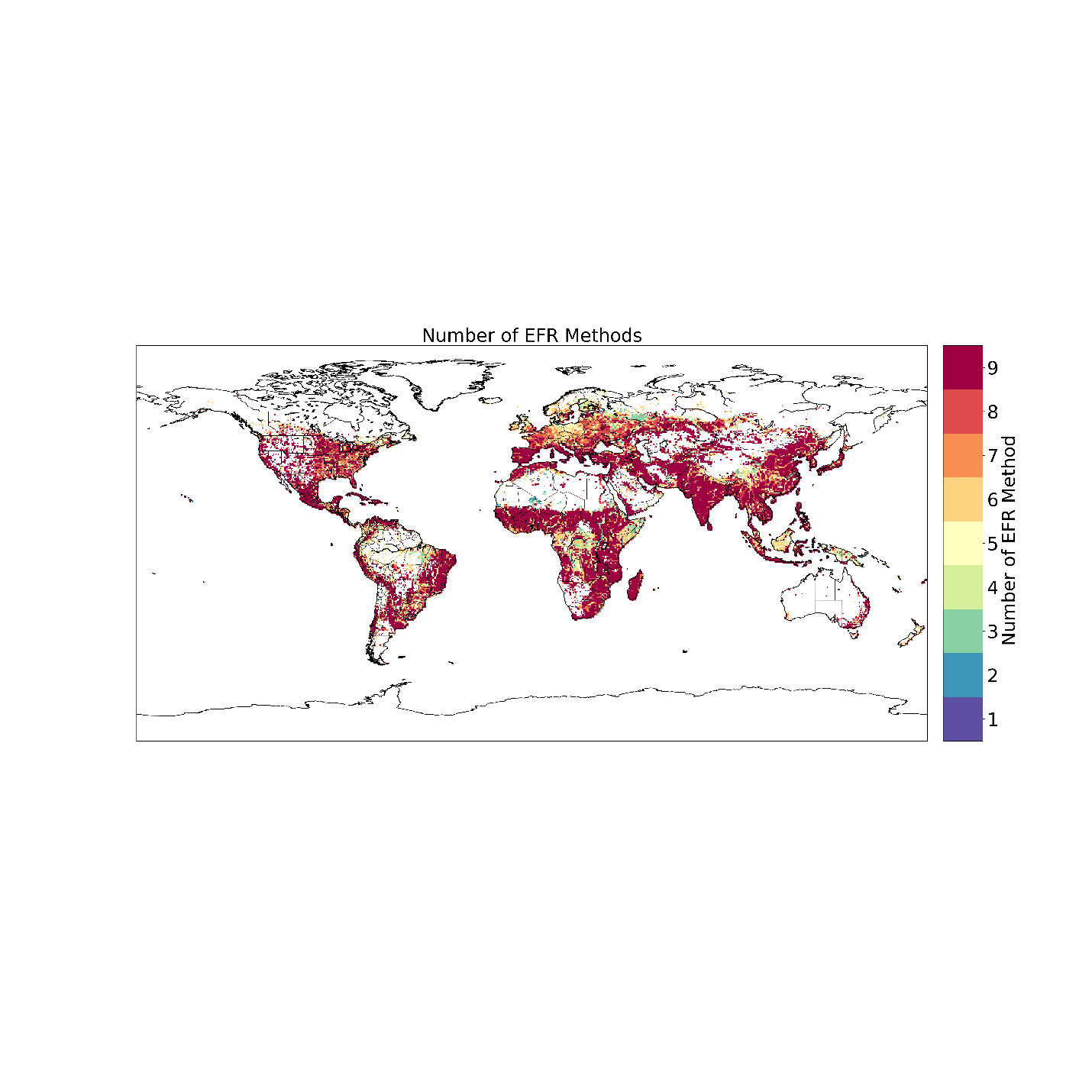   1. water scarcity at least one month a year   Figure S1: Spatial distribution of number of EFR methods estimating water scarcity in grids a) average of monthly water scarcity b) water scarcity at least one month a year (No EFR, 80% of Natural flow, VMF, Q90_Q50, Tessmann, Tennant, Smakhtin, MQ90, MMF). Grids with number of people greater than 1 person per Km^2^ are shown. |
| 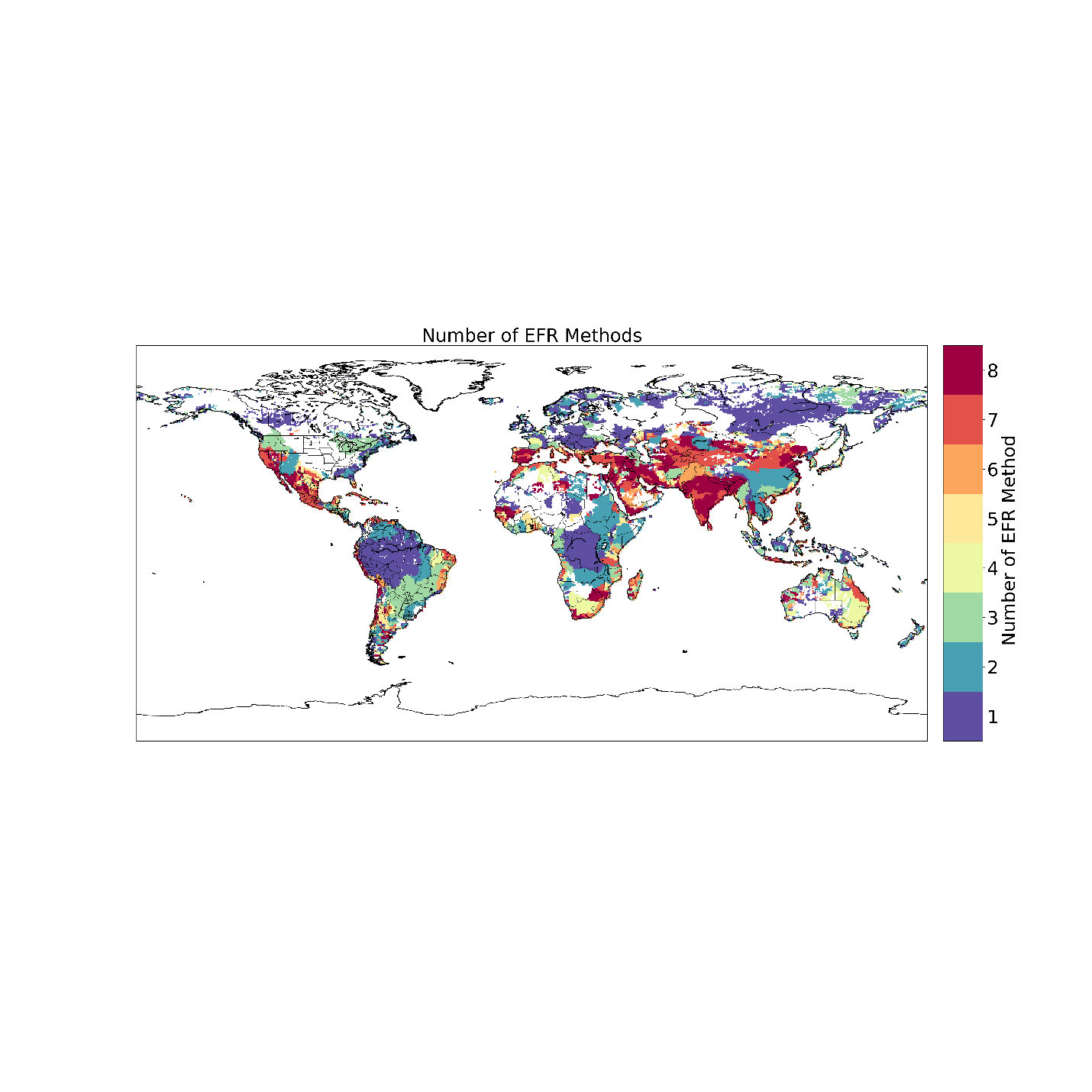   1. average of monthly water scarcity |
| 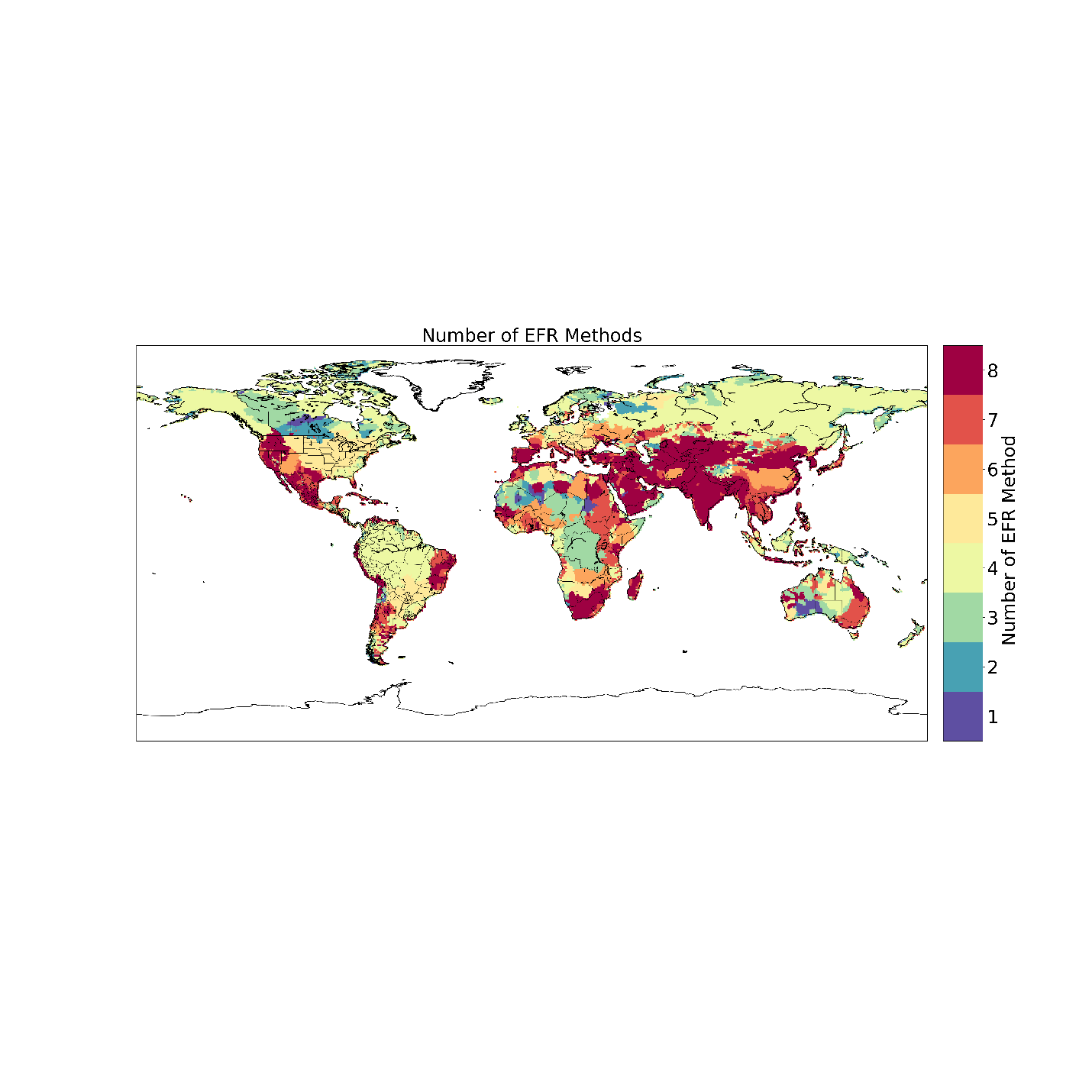   1. water scarcity at least one month a year   Figure S2: Spatial distribution of number of EFR methods estimating water scarcity in basins a) average of monthly water scarcity b) water scarcity at least one month a year (No EFR, MQ90, 80% of natural flow, VMF, Q90_Q50, Tessmann, Tennant, Smakhtin) |


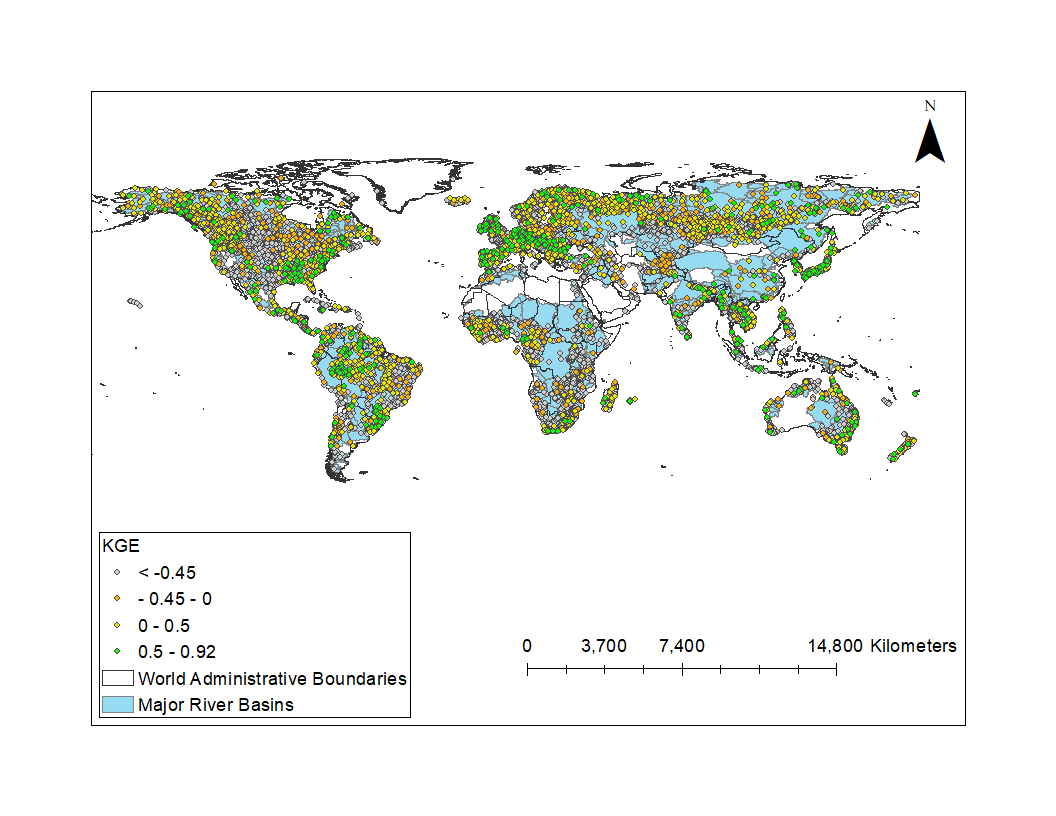


a) Location of stations used for validation of CWatM. The CWatM model used in this study resulted in monthly KGE > 0 on 1986 stations, monthly KGE > 0.5 on 552 stations having data from 1931-2019.


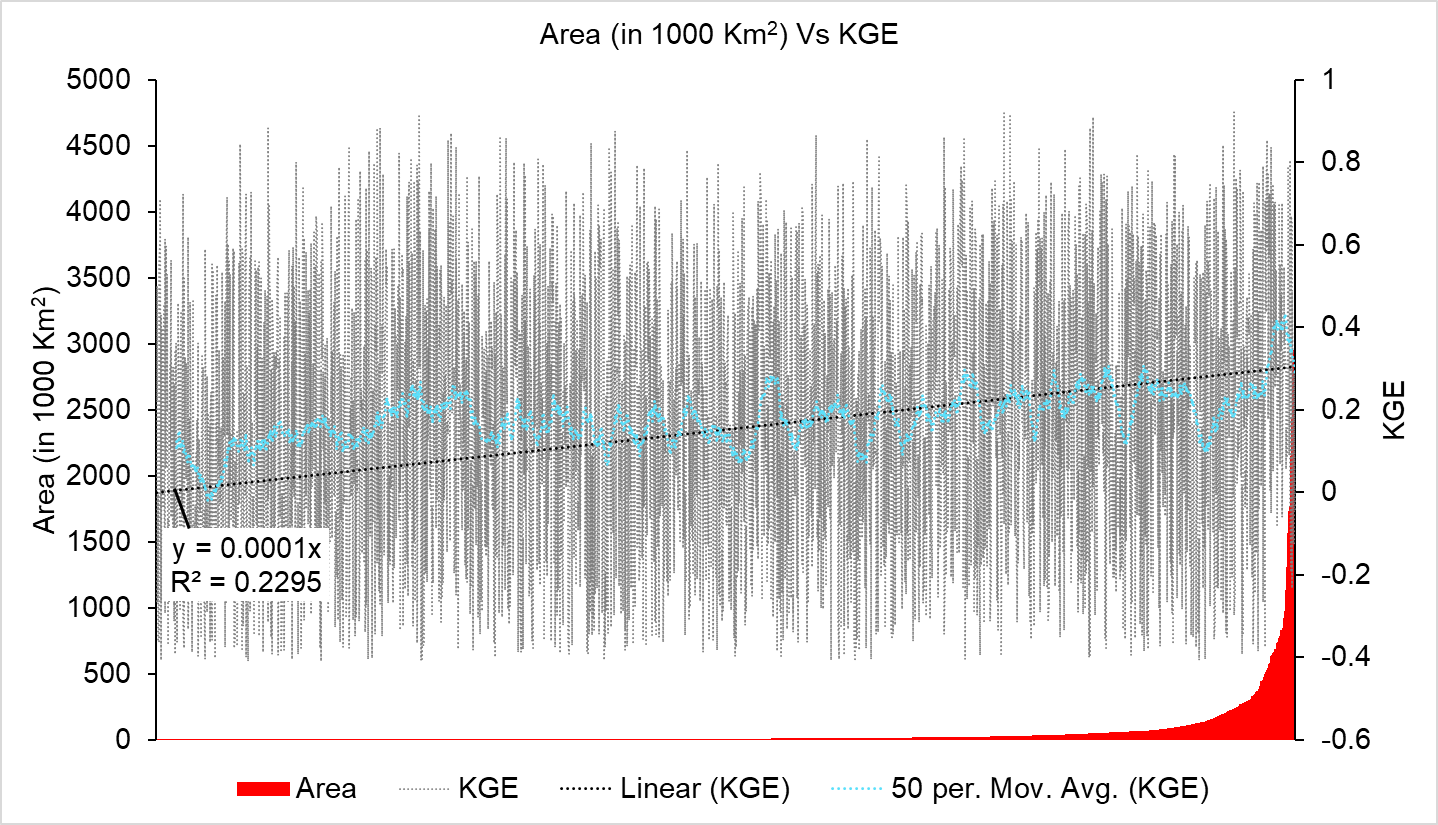


b) Correlation between basin area and KGE. KGE less than negative -0.45 is set to be -0.45 for fair display.


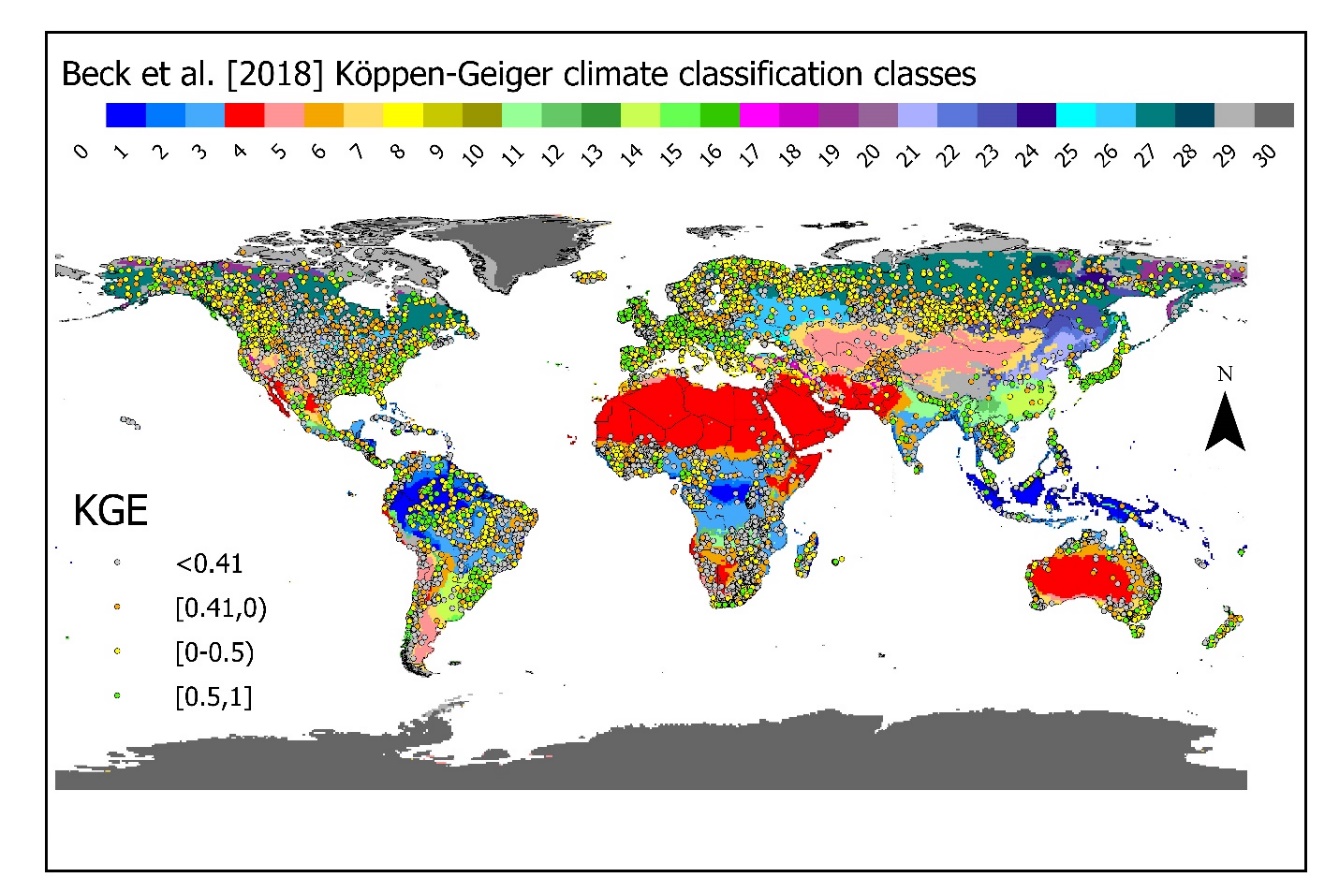

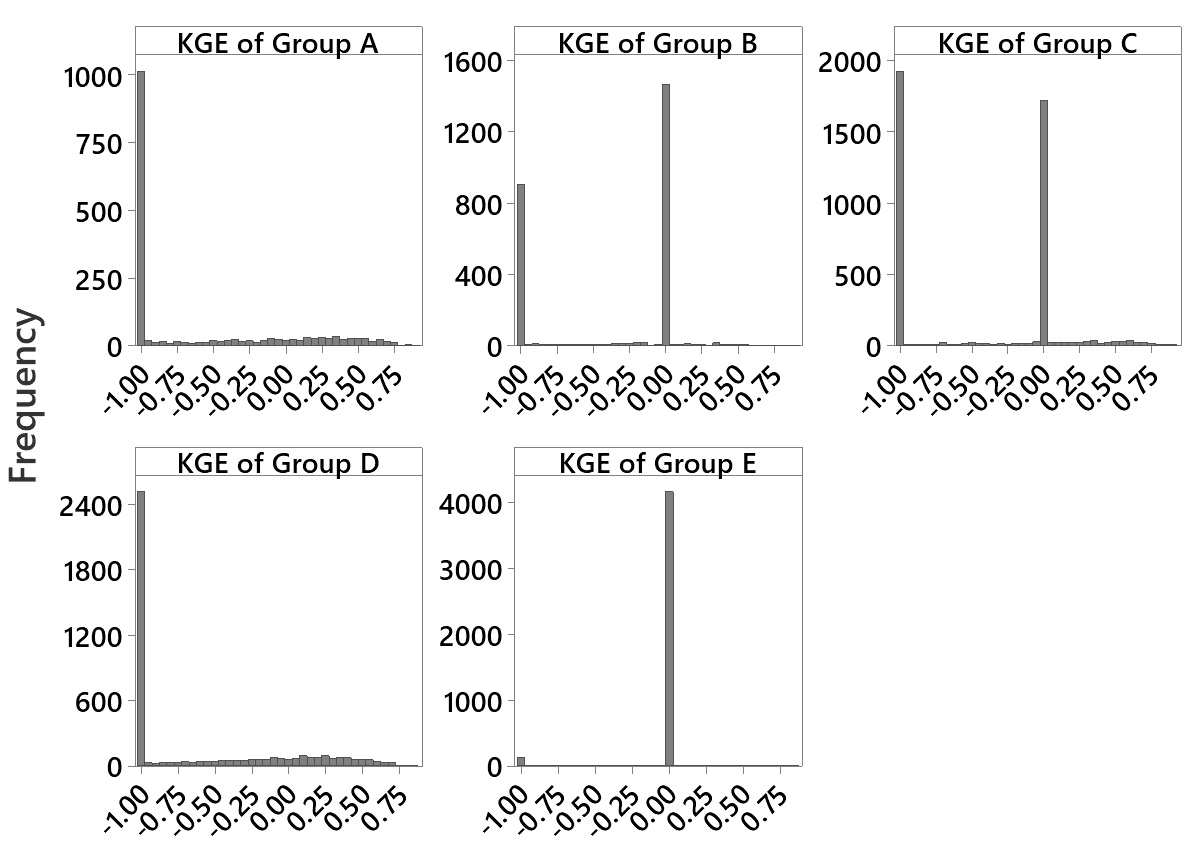

1. Correlation between climate zone and KGE.

| **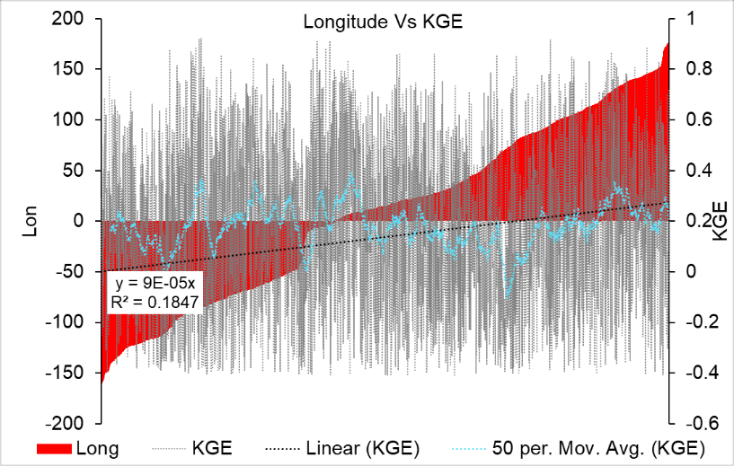** | | | **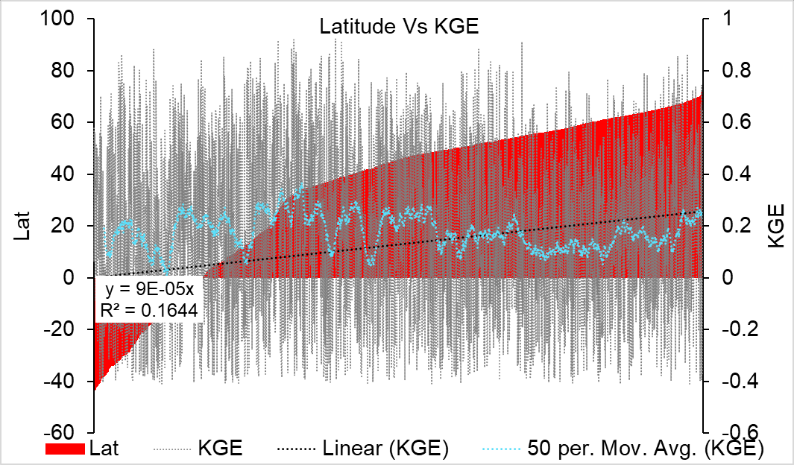** | |
| --- | --- | --- | --- | --- |
| 1. Correlation between Longitude and latitude with KGE   Figure S3: Evaluation of CWatM performance a) Location of stations used for validation b) Basin area, c) climate zone d) longitude and latitude. The model captured most of the global large basins. The KGE value is seen increasing with basin area in both linear trend line and moving average. Better KGE is found in Arid, Temperate and Polar zone as compared to other climate zones | | | | |
| 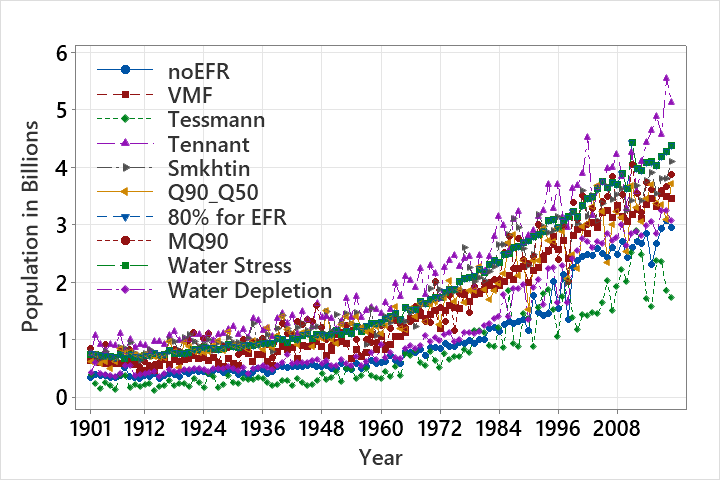  Figure S4: annual from monthly WSI comparison Basin | 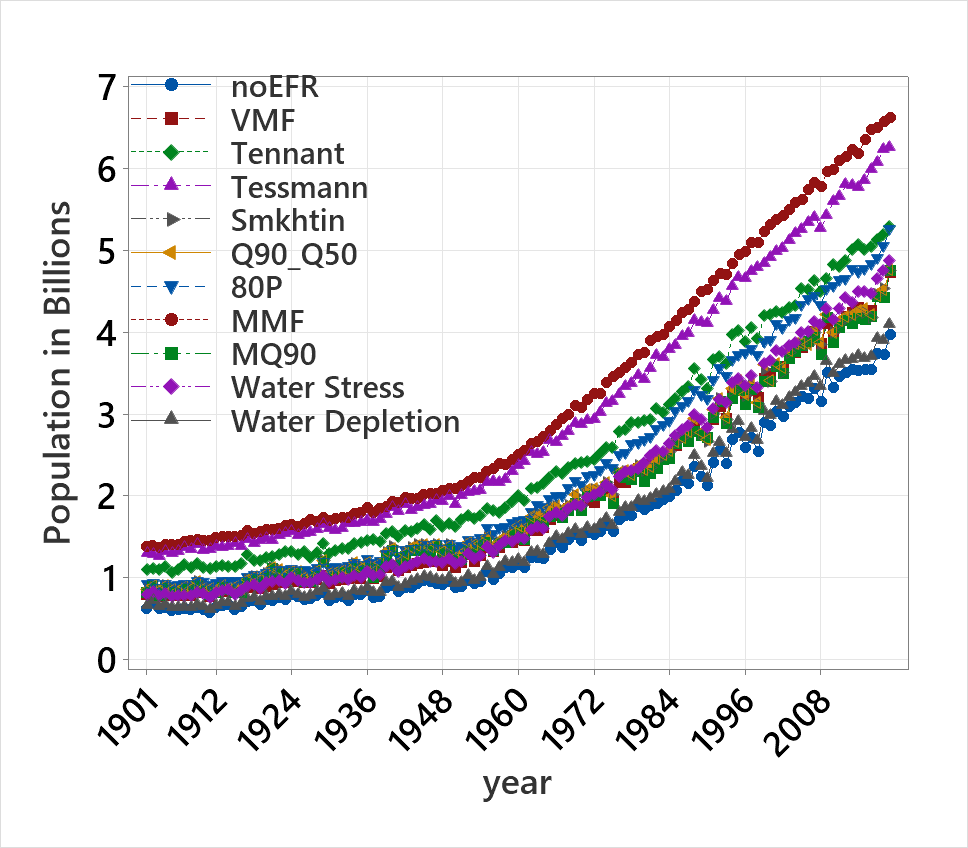  Figure S5: annual from monthly WSI comparison Grid | |  |  |
| 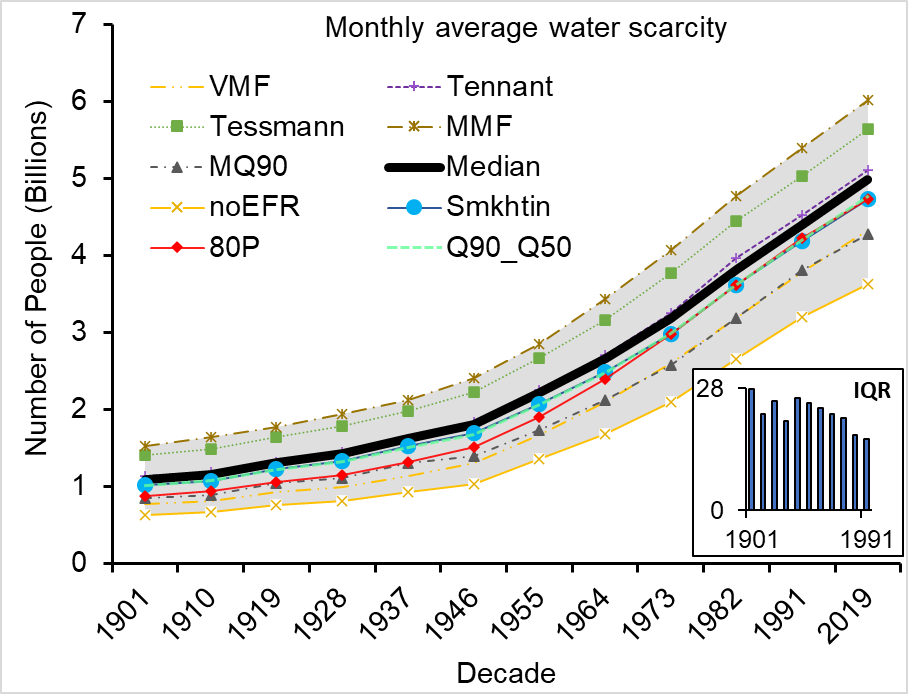 | 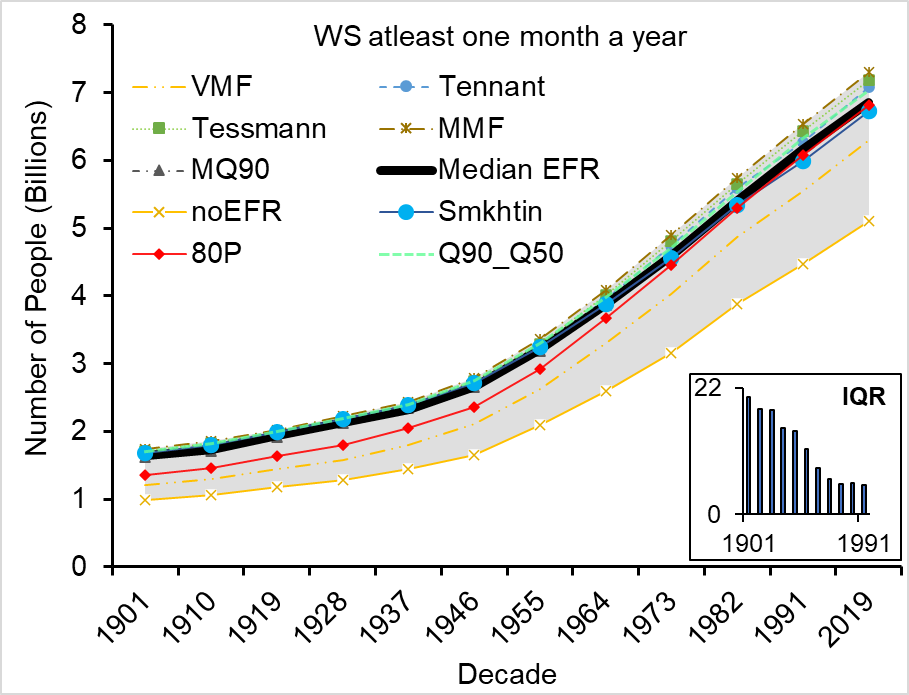 | |  |  |
| *Figure S6: Comparison between estimated number of people exposed to water scarcity using different EFR methods. Variable monthly flow (VMF), Tessmann, MQ90, noEFR, 80 percent of natural flow, Tennant, mean monthly flow (MMF), Smakhtin, Q90_Q50, and median EFR* | | |  |  |
| 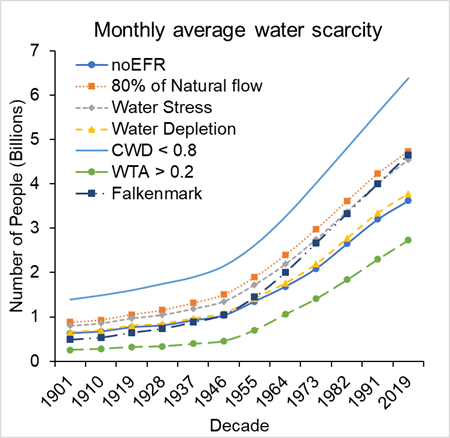 | 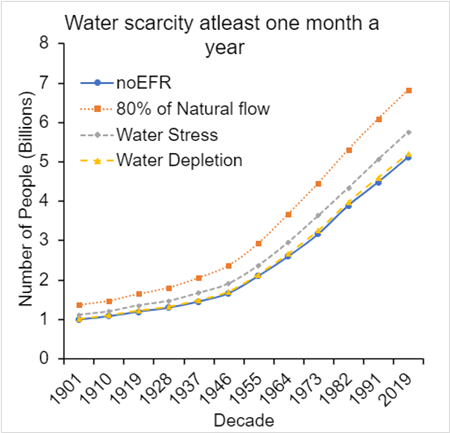 | |  |  |
| *Figure S7: Water scarcity trends 1901-2019. Water scarcity considering no EFR and 80% of natural flow as EFR, water stress, water depletion, CWD, WTA, and Falkenmark. CWD, WTA and Falkenmark values are the same for both plots therefore are shown only in one of the plots. a) Monthly average water scarcity b) Water scarcity at least one month a year* | | |  |  |

**
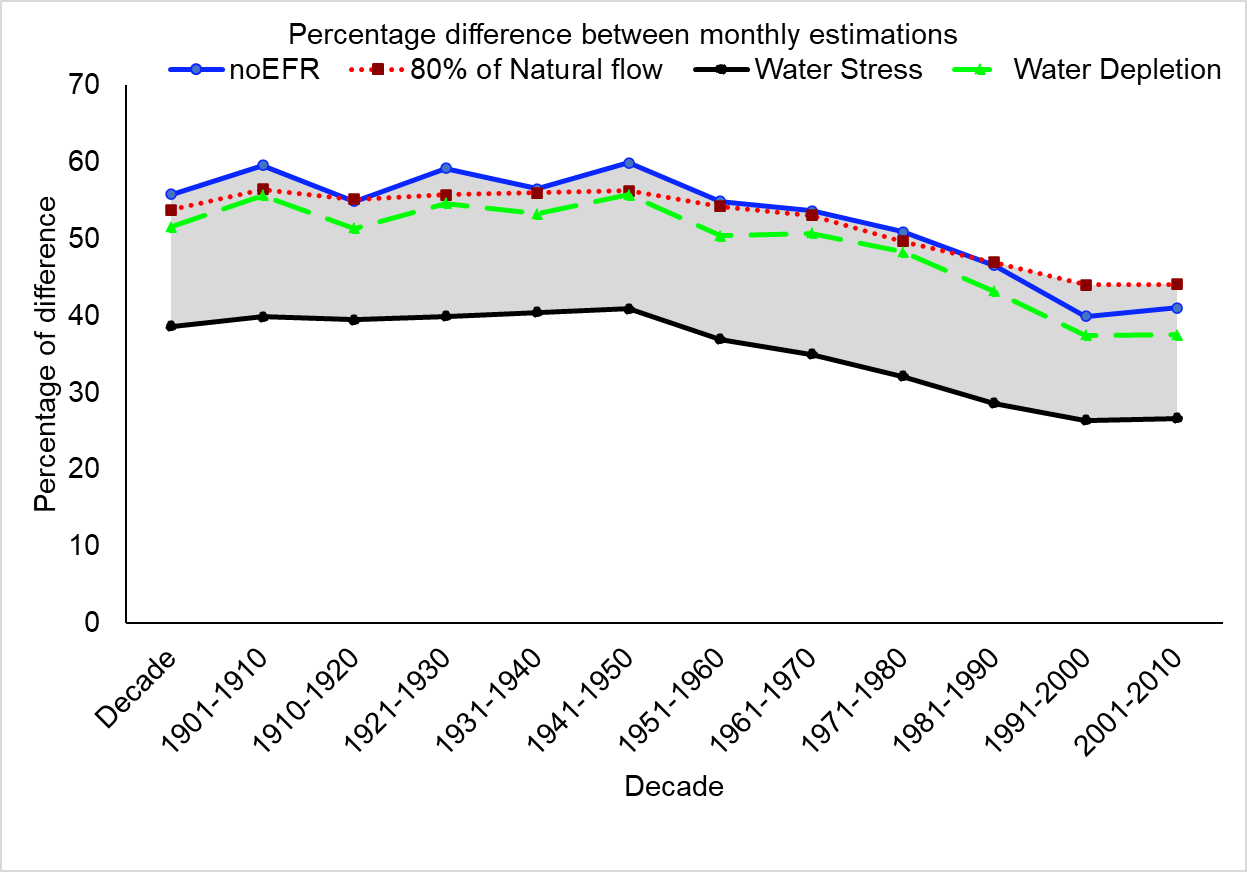
**

1. *Percentage difference between monthly average and water scarcity atleast one month a year 1901-2019*

| 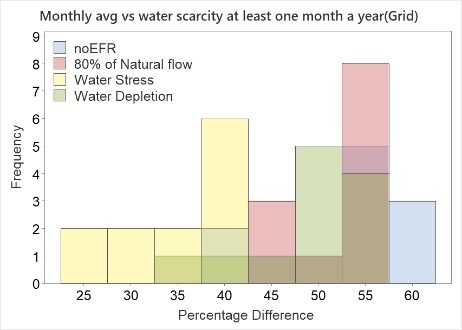 | 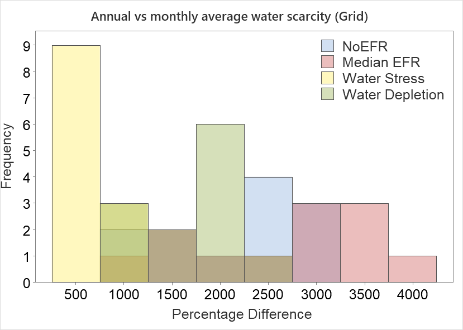 | 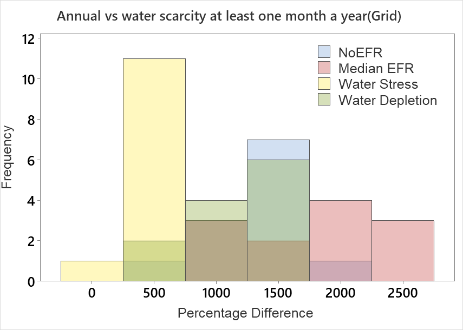 |
| --- | --- | --- |
| 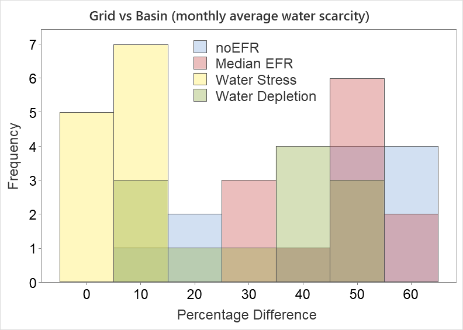 | 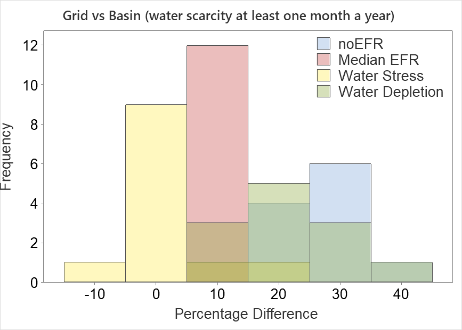 | 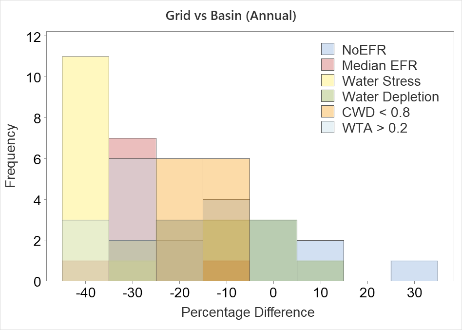 |

1. *Histograms of Percentage of difference (1901-2019)*

*Figure S8: Percentage of difference between a) monthly average water scarcity and water scarcity at least one month a year b) Histograms of Percentage of difference (1901-2019)*

**
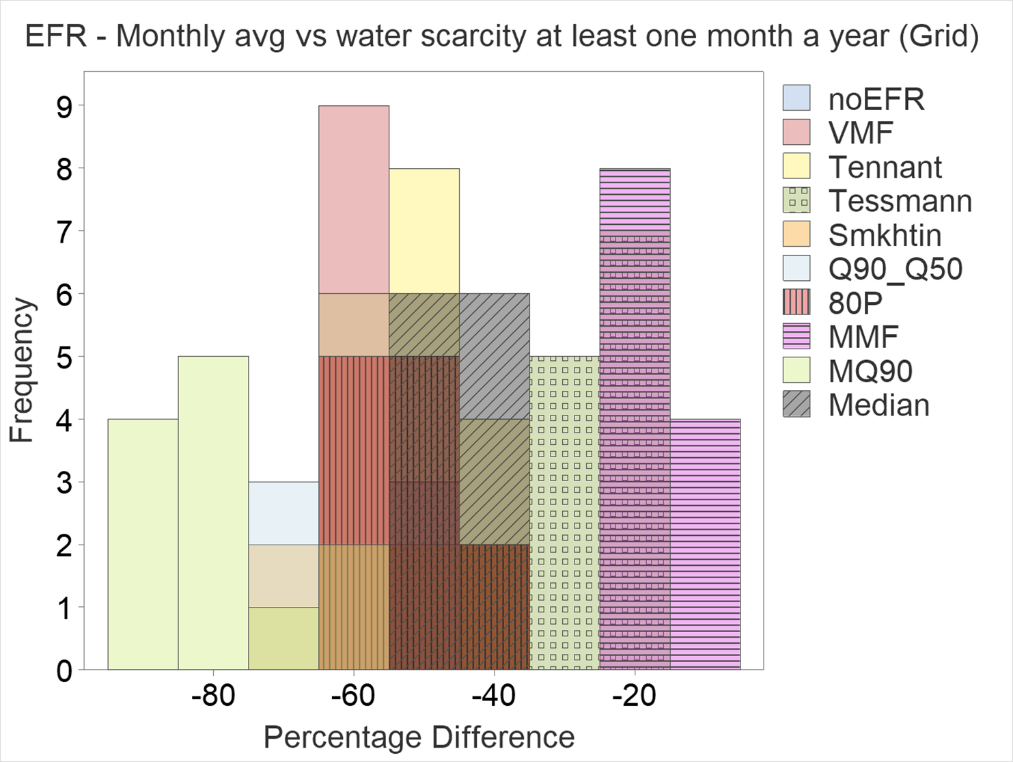
**

*Figure S9: Percentage of difference between a) monthly average water scarcity and water scarcity at least one month a year after accounting for EFR*

**
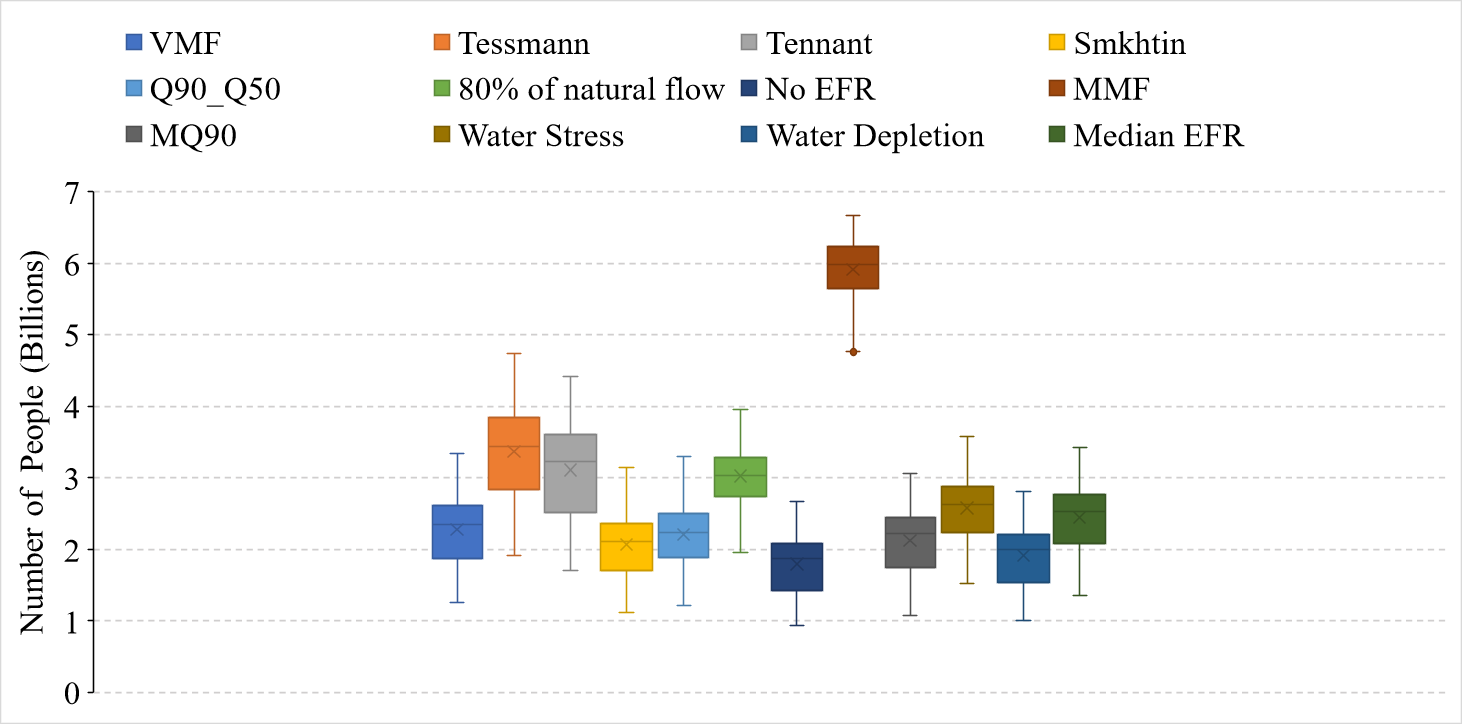
**

**
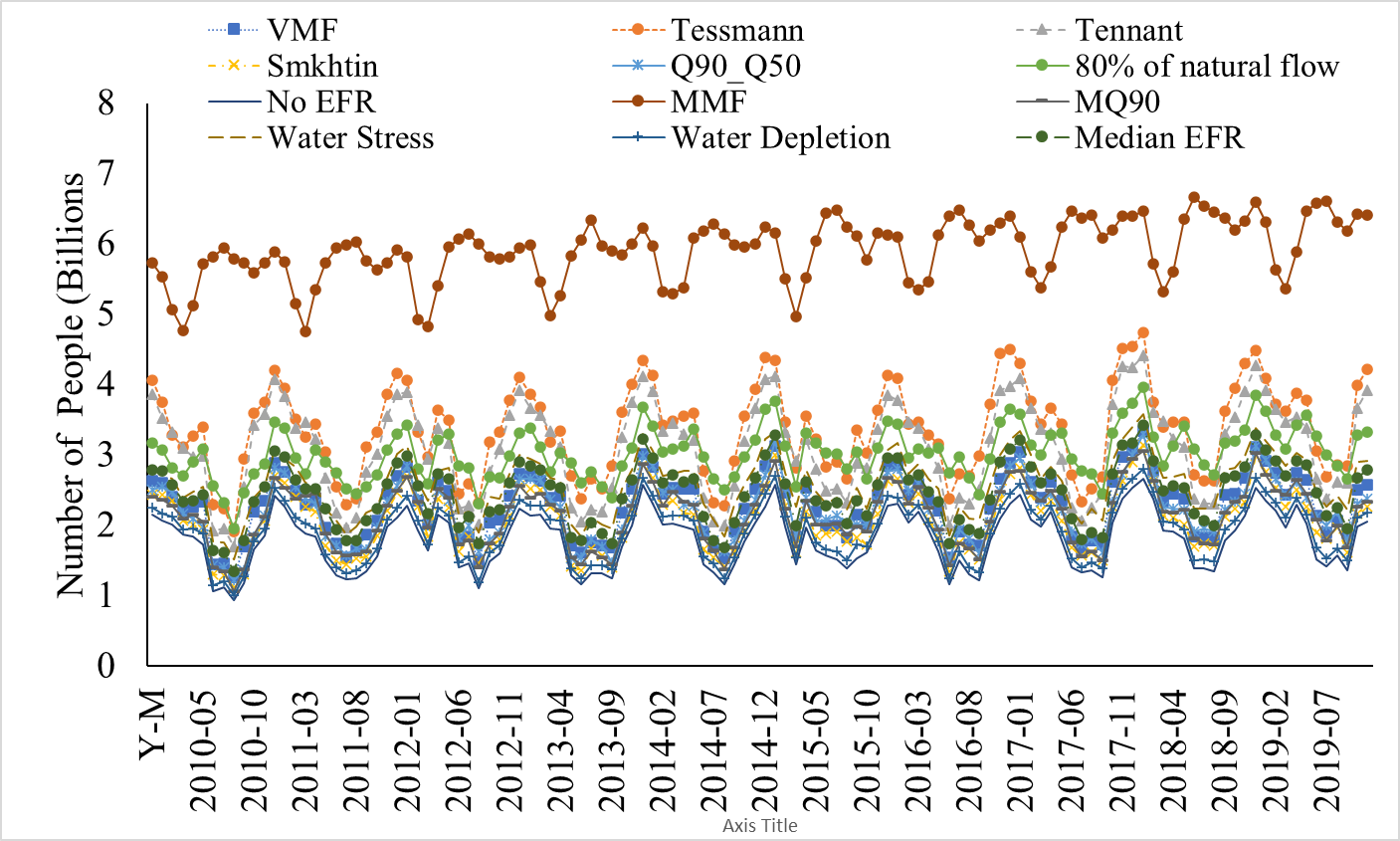
**

*a) Box plots of monthly water scarcity values on the grid level (2010-2019)*

**
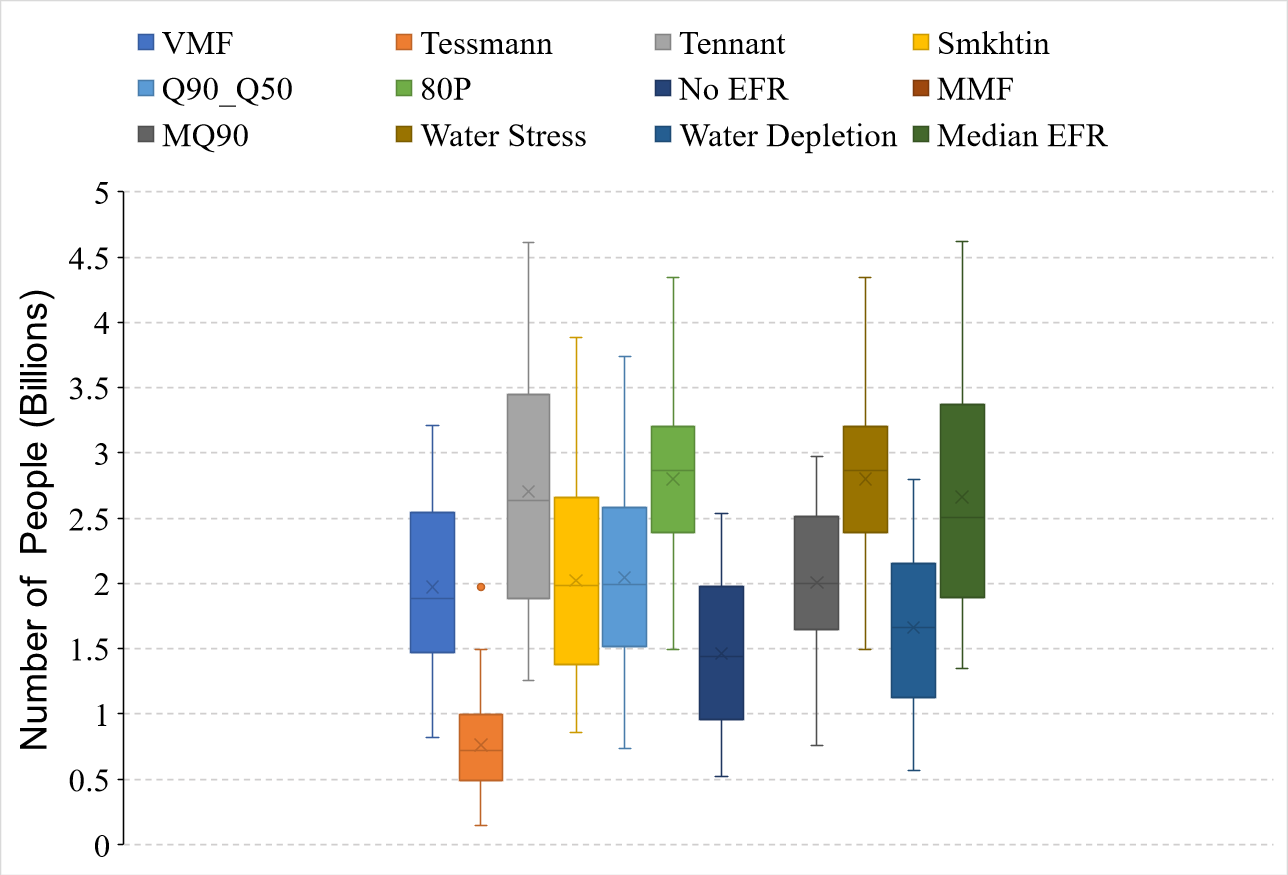

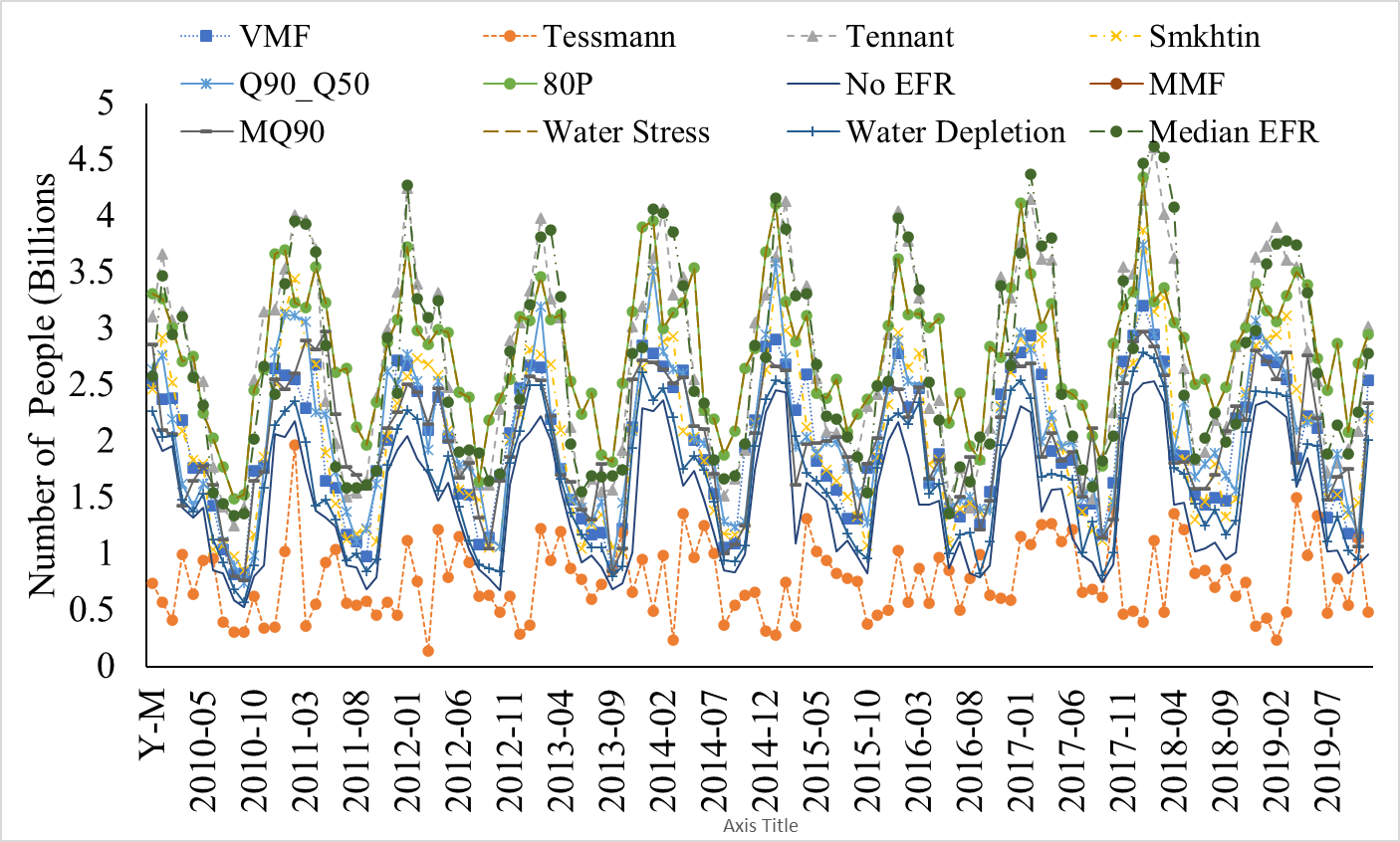
**

1. *Box plots of monthly water scarcity values on the basin level (2010-2019)*

*Figure S10: Box plots and time series of monthly water scarcity values on the basin level (2010-2019) a) at grid level b) at basin level.*

| 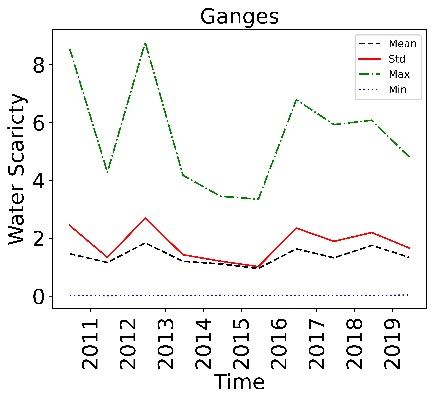 | 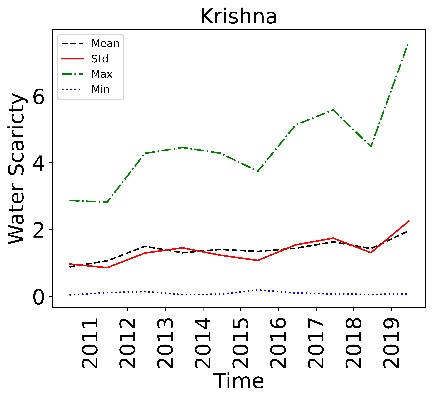 | 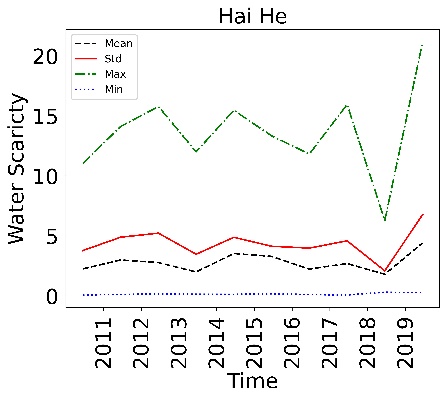 |
| --- | --- | --- |
| 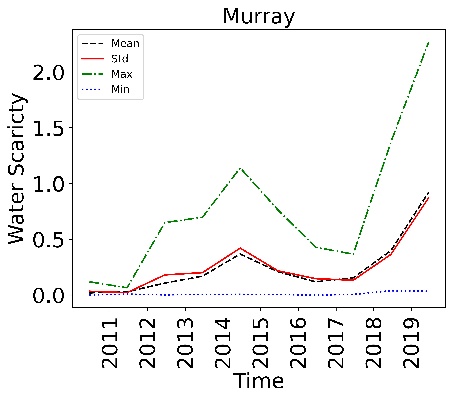 | 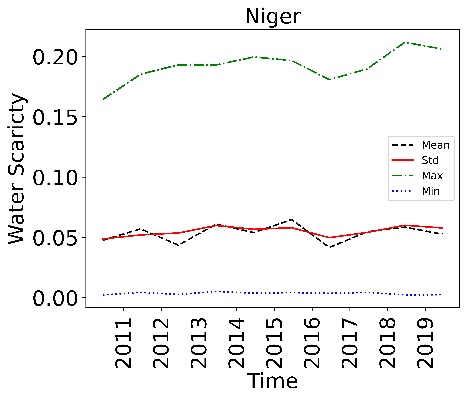 | 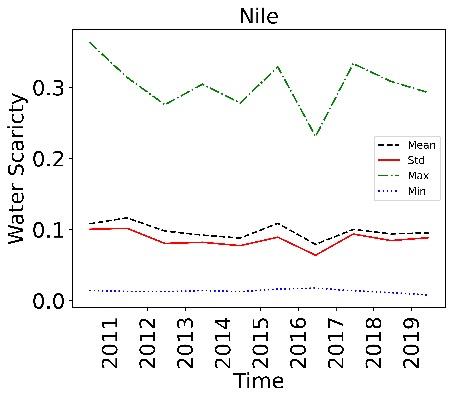 |
| 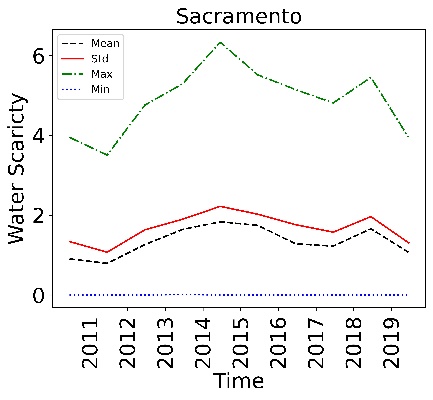 | 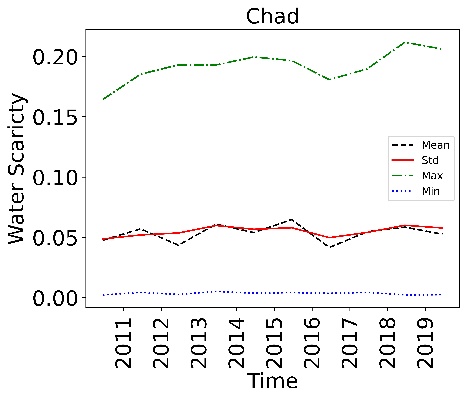 | 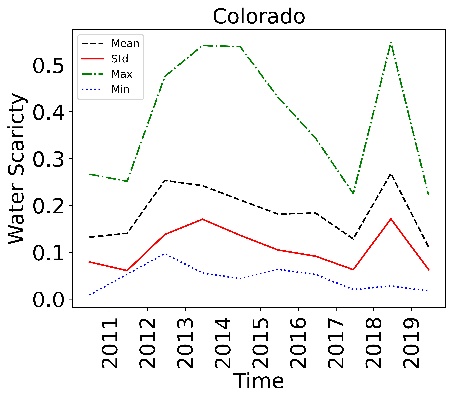 |

*Figure S11: Mean, standard deviation minimum, and maximum water scarcity values in 9 basins.*

**Reference Used**

Alcamo, J., DÖLl, P., Henrichs, T., Kaspar, F., Lehner, B., RÖSch, T., & Siebert, S. (2003). Development and testing of the WaterGAP 2 global model of water use and availability. *Hydrological Sciences Journal*, *48*(3), 317-337. <https://doi.org/10.1623/hysj.48.3.317.45290>

Arnell, N. W. (2004). Climate change and global water resources: SRES emissions and socio-economic scenarios. *Global Environmental Change-Human and Policy Dimensions*, *14*(1), 31-52. <https://doi.org/10.1016/j.gloenvcha.2003.10.006>

Brauman, K. A., Richter, B. D., Postel, S., Malsy, M., & Florke, M. (2016). Water depletion: An improved metric for incorporating seasonal and dry-year water scarcity into water risk assessments. *Elementa-Science of the Anthropocene*, *4*. <https://doi.org/ARTN> 000083

10.12952/journal.elementa.000083

Degefu, D. M., He, W. J., Liao, Z. Y., Yuan, L., Huang, Z. W., & An, M. (2018). Mapping Monthly Water Scarcity in Global Transboundary Basins at Country-Basin Mesh Based Spatial Resolution. *Scientific Reports*, *8*. <https://doi.org/ARTN> 2144

10.1038/s41598-018-20032-w

Doll, P., & Lehner, B. (2002). Validation of a new global 30-min drainage direction map. *Journal of Hydrology*, *258*(1-4), 214-231. <https://doi.org/Doi> 10.1016/S0022-1694(01)00565-0

Elvidge, C. D., Tuttle, B. T., Sutton, P. S., Baugh, K. E., Howard, A. T., Milesi, C., Bhaduri, B. L., & Nemani, R. (2007). Global distribution and density of constructed impervious surfaces. *Sensors*, *7*(9), 1962-1979. <https://doi.org/DOI> 10.3390/s7091962

Falkenmark, M. (1997). Meeting water requirements of an expanding world population. *Philosophical Transactions of the Royal Society of London Series B-Biological Sciences*, *352*(1356), 929-936. <https://doi.org/DOI> 10.1098/rstb.1997.0072

Falkenmark, M. (2013). Growing water scarcity in agriculture: future challenge to global water security. *Philosophical Transactions of the Royal Society a-Mathematical Physical and Engineering Sciences*, *371*(2002). <https://doi.org/ARTN> 20120410

10.1098/rsta.2012.0410

Falkenmark, M., Lundqvist, J., & Widstrand, C. (1989). Macro-Scale Water Scarcity Requires Micro-Scale Approaches - Aspects of Vulnerability in Semi-Arid Development. *Natural Resources Forum*, *13*(4), 258-267. <https://doi.org/DOI> 10.1111/j.1477-8947.1989.tb00348.x

GRDC. (2007). *GRDC Data Poratal/Global Runoff Data Centre*. <https://portal.grdc.bafg.de/applications/public.html?publicuser=PublicUser#dataDownload/Home>

Hanasaki, N., Kanae, S., Oki, T., Masuda, K., Motoya, K., Shirakawa, N., Shen, Y., & Tanaka, K. (2008). An integrated model for the assessment of global water resources Part 2: Applications and assessments. *Hydrology and Earth System Sciences*, *12*(4), 1027-1037. <https://doi.org/DOI> 10.5194/hess-12-1027-2008

Hanasaki, N., Yoshikawa, S., Pokhrel, Y., & Kanae, S. (2018). A Quantitative Investigation of the Thresholds for Two Conventional Water Scarcity Indicators Using a State-of-the-Art Global Hydrological Model With Human Activities. *Water Resources Research*, *54*(10), 8279-8294. <https://doi.org/https://doi.org/10.1029/2018WR022931>

Hansen, M. C., Potapov, P. V., Moore, R., Hancher, M., Turubanova, S. A., Tyukavina, A., Thau, D., Stehman, S. V., Goetz, S. J., Loveland, T. R., Kommareddy, A., Egorov, A., Chini, L., Justice, C. O., & Townshend, J. R. G. (2013). High-Resolution Global Maps of 21st-Century Forest Cover Change. *Science*, *342*(6160), 850-853. <https://doi.org/10.1126/science.1244693>

ISIMIP3a. (2021). <https://files.isimip.org/ISIMIP3a/InputData/climate/atmosphere/obsclim/global/daily/historical/GSWP3-W5E5/>

ISIMIP3a. (2022). <https://files.isimip.org/ISIMIP3a/>

Kummu, M., Ward, P. J., de Moel, H., & Varis, O. (2010). Is physical water scarcity a new phenomenon? Global assessment of water shortage over the last two millennia. *Environmental Research Letters*, *5*(3). <https://doi.org/Artn> 034006

10.1088/1748-9326/5/3/034006

Lehner, B., Liermann, C. R., Revenga, C., Vorosmarty, C., Fekete, B., Crouzet, P., Doll, P., Endejan, M., Frenken, K., Magome, J., Nilsson, C., Robertson, J. C., Rodel, R., Sindorf, N., & Wisser, D. (2011). High-resolution mapping of the world's reservoirs and dams for sustainable river-flow management. *Frontiers in Ecology and the Environment*, *9*(9), 494-502. <https://doi.org/10.1890/100125>

Liu, X. C., Liu, W. F., Liu, L., Tang, Q. H., Liu, J. G., & Yang, H. (2021). Environmental flow requirements largely reshape global surface water scarcity assessment. *Environmental Research Letters*, *16*(10). <https://doi.org/ARTN> 104029

10.1088/1748-9326/ac27cb

Mekonnen, M. M., & Hoekstra, A. Y. (2016). Four billion people facing severe water scarcity. *Sci Adv*, *2*(2), e1500323. <https://doi.org/10.1126/sciadv.1500323>

Messager, M. L., Lehner, B., Grill, G., Nedeva, I., & Schmitt, O. (2016). Estimating the volume and age of water stored in global lakes using a geo-statistical approach. *Nature Communications*, *7*. <https://doi.org/ARTN> 13603

10.1038/ncomms13603

Oki, T., Agata, Y., Kanae, S., Saruhashi, T., Yang, D. W., & Musiake, K. (2001). Global assessment of current water resources using total runoff integrating pathways. *Hydrological Sciences Journal-Journal Des Sciences Hydrologiques*, *46*(6), 983-995. <https://doi.org/Doi> 10.1080/02626660109492890

Pastor, A. V., Ludwig, F., Biemans, H., Hoff, H., & Kabat, P. (2014). Accounting for environmental flow requirements in global water assessments. *Hydrology and Earth System Sciences*, *18*(12), 5041-5059. <https://doi.org/10.5194/hess-18-5041-2014>

Portmann, F. T., Siebert, S., & Doll, P. (2010). MIRCA2000-Global monthly irrigated and rainfed crop areas around the year 2000: A new high-resolution data set for agricultural and hydrological modeling. *Global Biogeochemical Cycles*, *24*. <https://doi.org/Artn> Gb1011

10.1029/2008gb003435

Richter, B. D., Davis, M. M., Apse, C., & Konrad, C. (2012). A Presumptive Standard for Environmental Flow Protection. *River Research and Applications*, *28*(8), 1312-1321. <https://doi.org/10.1002/rra.1511>

Smakhtin, V., Revenga, C., & Doll, P. (2004). A pilot global assessment of environmental water requirements and scarcity. *Water International*, *29*(3), 307-317. <https://doi.org/Doi> 10.1080/02508060408691785

Tennant, D. (1976). Instream Flow Regimens for Fish, Wildlife, Recreation and Related Environmental Resources. *Fisheries*, *1*, 6-10. <https://doi.org/10.1577/1548-8446(1976)001><0006:IFRFFW>2.0.CO;2

Tessmann. (1980). In *Environmental Assessment, Technical Appendix E in Environmental Use Sector Reconnaissance Elements of the Western Dakotas Region of South Dakota Study*. Water Resources Research Institute South Dakota State University.

Vorosmarty, C. J., Green, P., Salisbury, J., & Lammers, R. B. (2000). Global water resources: Vulnerability from climate change and population growth. *Science*, *289*(5477), 284-288. <https://doi.org/DOI> 10.1126/science.289.5477.284

Wada, Y., Van Beek, L. P. H., Viviroli, D., Durr, H. H., Weingartner, R., & Bierkens, M. F. P. (2011). Global monthly water stress: 2. Water demand and severity of water stress. *Water Resources Research*, *47*. <https://doi.org/Artn> W07518

10.1029/2010wr009792

Zhang, Y. G., & Schaap, M. G. (2017). Weighted recalibration of the Rosetta pedotransfer model with improved estimates of hydraulic parameter distributions and summary statistics (Rosetta3). *Journal of Hydrology*, *547*, 39-53. <https://doi.org/10.1016/j.jhydrol.2017.01.004>

1. EFR: Environmental flow requirement [↑](#footnote-ref-2)
2. RWS: Water scarcity index [↑](#footnote-ref-3)
3. WSI: Water scarcity indicator [↑](#footnote-ref-4)
4. River withdrawal = channel abstraction [↑](#footnote-ref-5)
5. LFR = Low Flow Requirement [↑](#footnote-ref-6)
6. HFR=High Flow Requirement [↑](#footnote-ref-7)
7. IFR= Intermediate Flow Requirement [↑](#footnote-ref-8)
